# Towards Sensitization Profiling for Allergy Prevention in Russia: A Systematic Review

**DOI:** 10.3390/ijms27125334

**Published:** 2026-06-12

**Authors:** Alexandra Dubovets, Anastasia Lukashevichus, Valery Artemova, Olga Belik, Daria Trifonova, Irina Evsegneeva, Alexander Karaulov, Inna Tulaeva

**Affiliations:** 1 Laboratory of Immunopathology, Department of Clinical Immunology and Allergology, I.M. Sechenov First Moscow State Medical University, Moscow 119992, Russia; 2 Life Improvement by Future Technologies (LIFT) Center, Moscow 143025, Russia; 3 Institute of Pathophysiology and Allergy Research, Center for Pathophysiology, Infectiology and Immunology, Medical University of Vienna, 1090 Vienna, Austria

**Keywords:** allergen, allergic sensitization, IgE profile, respiratory allergy, preventive allergen-specific immunization

## Abstract

Allergy is the most common hypersensitivity disorder, affecting around 30% of the global population. Due to its rapidly increasing prevalence and significant reduction in quality of life for patients, allergy represents a major public health problem, and the improvement of diagnostic and treatment options for allergic diseases is of utmost importance. Moreover, the development of preventive allergen-specific immunization strategies is an emerging research direction in mitigating allergy incidence, especially for respiratory diseases such as allergic rhinitis and asthma. Since environmental allergen exposures differ substantially depending on climatogeographical, ecological, and behavioral factors, investigating local IgE sensitization profiles could significantly contribute to optimizing allergy management. We performed a systematic database review to summarize available knowledge on IgE sensitization profiles in Russia across different regions of the country. The study was conducted in compliance with PRISMA and SWiM guidelines and registered in the PROSPERO database (CRD420250650847). We identified major differences in sensitization profiles across certain geographical areas, reported in 60 studies. However, heterogeneity of methods and gaps in the existing evidence were noted, and, as the available data appear insufficient for reliable profiling, an outline was proposed for systematic and methodologically harmonized studies necessary to develop further region-tailored approaches.

## 1. Introduction

The spectrum of allergic diseases includes allergic rhinitis and asthma, atopic dermatitis, and food allergies. The prevalence of atopic disorders has risen substantially in recent decades, and it is expected that by 2050, more than 4 billion will suffer from allergies [1]. In the 1990s, epidemiological studies had already shown that the prevalence of allergic diseases in Russia was 15–35% in adults and 15–20% in children, which is in line with the data obtained in westernized countries; more recent studies report the presence of atopy in more than 30% of children [2,3]. Respiratory allergy is the predominant pathology in adolescents and adults, and airborne allergens are the most difficult to avoid; thus, inhalant allergens such as pollen and indoor allergens remain in the focus of allergen-specific immunotherapy (AIT) developments [4,5]. Among non-respiratory allergens, peanuts are an example of a complex allergen source with a high incidence of life-threatening anaphylaxis often accompanied by asthma [6]. Given the large proportion of the population affected and the complexity and clinical diversity of allergic diseases, there is a need to advance not only diagnostic and therapeutic strategies but also long-term effective prophylactic approaches targeting the pathogenesis of allergy.

**Figure 1:**
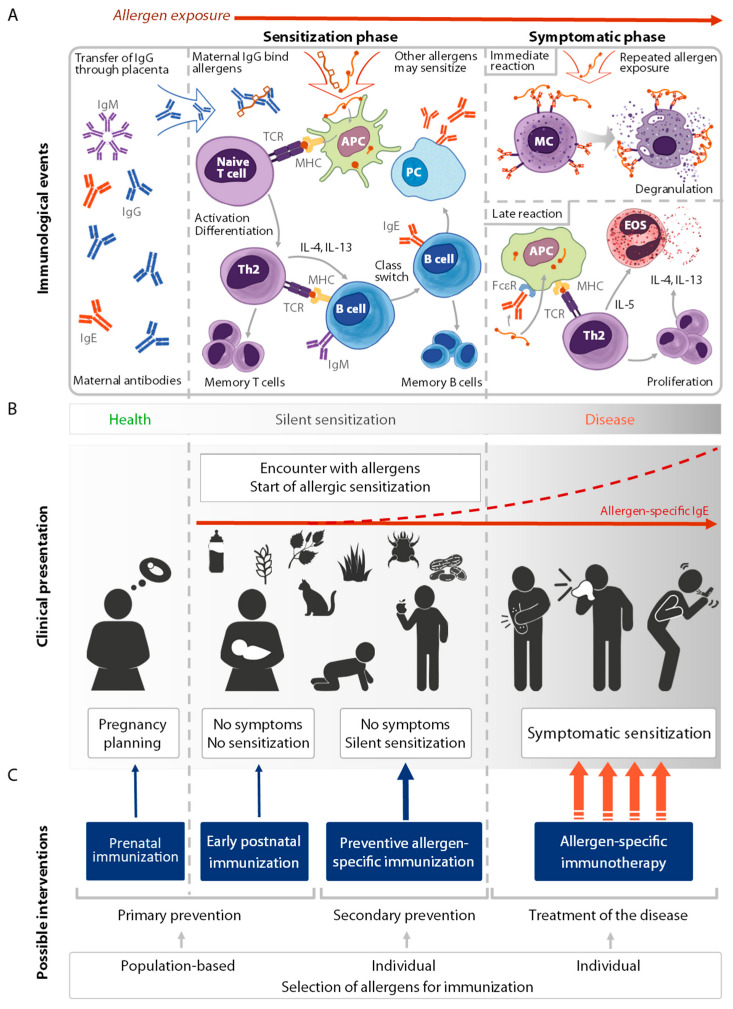
**Allergy development and possible introduction of allergen-specific therapy and prophylaxis.** (**A**) IgE-dependent allergic diseases develop through a series of coordinated immunological events that begin with allergen sensitization. Importantly, maternal allergen-specific IgG transferred transplacentally may bind allergens during the first months of life and influence the infant’s initial immune responses. During the sensitization phase, APCs capture and process allergens and present them via MHC to naïve T cells, which develop a Th2 phenotype and promote the production of IL-4, IL-5, and IL-13. These cytokines induce B-cell class switching to IgE, which subsequently binds to high-affinity FcεRI receptors on mast cells and basophils. Upon later re-exposure to the allergen begins the symptomatic phase: cross-linking of receptor-bound IgE triggers mast cell degranulation and the release of proinflammatory mediators, leading to the immediate hypersensitivity reaction. Recruitment of eosinophils and other inflammatory cells contributes to the late-phase response and the chronic inflammation characteristic of persistent allergic disease. (**B**) Clinical manifestations of allergic reactions occur upon re-exposure to the allergen during the symptomatic phase, which is preceded by a period of silent sensitization beginning after birth with the first exposures to allergens. (**C**) Possible intervention points for allergen-specific prophylactic immunization include pre- and postnatal prophylactic immunization as options for primary prevention, secondary prevention during the silent sensitization phase, and finally AIT for symptomatic allergy. APC—antigen-presenting cell; FcεR—Fc epsilon receptor; Ig—immunoglobulin; IL—interleukin; MHC—major histocompatibility complex; TCR—T-cell receptor; Th2—T helper type 2 cell.

Immunoglobulin E (IgE) sensitization is the key pathophysiological mechanism of allergic diseases. The immunological events leading to allergy manifestation are depicted in Figure 1A. There are still gaps in the knowledge of how primary sensitization occurs; however, the available data suggests that this process most actively takes place in the first months or years of life [7,8,9,10]. The outcome of early exposure to allergens could be regulated by maternal antibodies that pass through the placenta to the child [11,12]. It is determined that IgG is the dominant class of antibodies that is actively transported across the placenta using the neonatal Fc receptor (FcRn) [13,14]. There is experimental evidence that maternal IgE potentially may also be transferred through the placenta, however these results were mostly generated using animal models and the previously obtained human data suggested otherwise [14,15]. Thus, the transfer of maternal IgG confers passive immunity to certain antigens, including allergens, and these antibodies may bind the respective allergens and thus prevent the IgE sensitization [12,16,17]. In the case of a lack of allergen-specific IgG, the allergen is taken up by antigen-presenting cells (APCs) that prime allergen-specific T cells and may then stimulate potent T helper 2 (Th2) responses. The microenvironment within the pulmonary airways is particularly conducive to Th2 response development [5,18,19]. Th2 cells produce cytokines, e.g., interleukin 4 (IL-4) and IL-13, that promote B cell activation and class switching towards IgE production. As a result of the sensitization phase, allergic individuals produce allergen-specific IgE and a pool of allergen-specific memory T and B cells [20,21].

After the sensitization phase, repeated allergen exposure triggers several effector mechanisms. IgE binds to mast cells and basophils via the high-affinity receptor FcεRI and, when cross-linked by the respective allergen, stimulates the release of pro-inflammatory substances such as histamine and leukotrienes, which results in an immediate inflammatory response. The late-phase reactions are characterized by T cell infiltration as a result of both IgE-dependent and independent APC-T cell interactions, and eosinophil activation depending mainly on IL-5 secretion [20]. A clinical reflection of the aforementioned immunological processes becomes evident rather late, with the increase in allergen-specific IgE levels (Figure 1B). The sensitization phase is asymptomatic, and only upon repeated allergen exposure, which may occur months or years after the first encounter, the disease manifestation could happen.

Accordingly, possible interventions that modify the pathological process (Figure 1C) can be divided into two categories: AIT—an administration of disease-causing allergens usually applied at the symptomatic stage when IgE-sensitization has become clinically evident, and preventive allergen-specific immunization, implemented before the first symptoms occur. AIT was efficient in patients with IgE-dependent allergic rhinitis in preventing the development of asthma [22,23]; when given continuously for several years, there is often a persistent clinical improvement for several years after its discontinuation [24], and it has been shown that the success of AIT is higher when it is initiated earlier [25]. This principle could be extrapolated to preventive allergen-specific immunization as a secondary prevention, i.e., when sensitization, or allergen-specific IgE, is already detectable, and primary prevention, i.e., before T cell priming occurs and the IgE response is established, which may have potential to be more efficient than AIT for symptomatic allergy [26]. Primary prevention can be carried out pre- or postnatally—for example, by immunizing the mother during pregnancy planning or later, in the newborn, in line with other standard vaccinations [27,28]. However, the evidence is yet limited to the prevention of new sensitizations in a small number of monosensitized children [29], and thus primary prevention remains to be a theoretical concept requiring a robust proof of principle.

In the context of preventive allergen-specific immunization, the careful selection of allergens based on the precise identification of the allergy culprit is of central importance. In situations where sensitization is already established—such as in manifest allergic disease or in secondary prevention—the choice of allergens is guided by the patient’s detectable allergen-specific IgE profile. In contrast, primary prevention presents a fundamentally different challenge: no sensitization has yet developed or can be detected. This necessitates a deliberate and evidence-based selection of allergens. To make such a selection and determine the demand for therapeutic and preventive molecular allergy vaccines, a thorough understanding of IgE sensitization profiles is essential for any future development of preventive concepts. Local sensitization profiles are driven by the totality of environmental exposures, called the exposome, and may differ even in geographically neighboring locations [30,31,32]. Russia encompasses numerous climate zones, ethnic groups, and environmental factors. Here, we aim to summarize existing knowledge on the relevance of different allergens in diverse areas of the country and identify unmet needs in sensitization profiling.

## 2. Materials and Methods

### 2.1. Protocol, Registration and Data Collection

**Figure 2:**
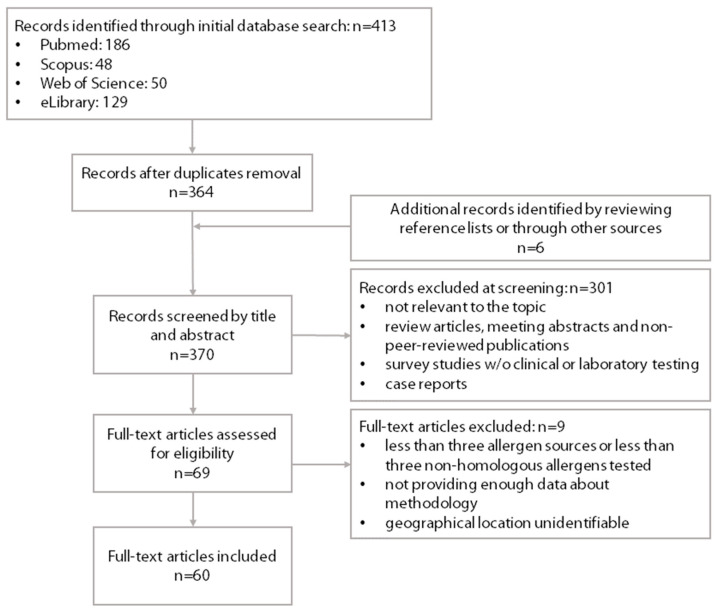
Flowchart of the study selection procedure.

In the present study, a meticulous search strategy was implemented across four electronic databases in order to analyze the relevant literature on local IgE sensitization profiles in various regions of Russia (Figure 2). The protocol for this systematic search was developed in accordance with the PRISMA (Preferred Reporting Items for Systematic Reviews and Meta-Analyses) and SWiM (Synthesis Without Meta-analysis) guidelines to ensure a structured, transparent and reproducible methodology [33,34]. Since the studies varied considerably in design and methodology, no quantitative synthesis through meta-analysis was conducted. To support open access to the research process and its results, the review was registered in the progressively registered systematic reviews database (PROSPERO, date of registration: 24 February 2025, registration number: CRD420250650847).

A systematic literature search was conducted in the PubMed, Scopus, Web of Science, and eLibrary databases. The search strategy included combinations of the following keywords or the respective Medical Subject Headings (MeSH) terms: “allergen”, “allergy”, “sensitization”, “sensibilization”, “profile”, “profiling”, “immunoglobulin E”, “IgE”, “skin test”, “allergens”, and “Russia.” Boolean operators (AND/OR) were applied to refine the search strategy. Filtering based on language restriction was not applied throughout the whole process. A manual screening of reference lists of the retrieved original articles and previous reviews was performed in order to identify additional potentially relevant studies.

### 2.2. Inclusion and Exclusion Criteria

The following inclusion criteria were used to assess the eligibility of the retrieved articles: publication date ranging from 1 January 2000 to 1 August 2025; reporting of the data on allergic sensitization assessed by skin testing and/or serological determination of sIgE presence; availability of data on at least three different non-cross-reactive allergens from at least three different allergen sources; and study conducted on participants/samples from Russia with known geographical origin. The following exclusion criteria were applied: non-peer-reviewed publications; survey studies without original data on skin or sIgE testing; lack of the information regarding the region where the study was conducted; and inadequate description of the study methodology. The studies were screened and the data were extracted by two authors independently, and in case of disagreements, a third author was consulted to reach the decision by a consensus.

### 2.3. Selection Process and Data Extraction

A total of 370 publications on the topic were analyzed, of which 60 publications met the inclusion criteria and were selected for further analysis. A PRISMA flow diagram illustrating the selection process was constructed to provide a transparent overview (Figure 2). The data were extracted in table format and included the first author’s last name, publication year, sample size, study population, age, sex, testing methods, number and type of components tested, and relevant sensitization prevalence. As a metric of relevant IgE sensitization, the results showing more than 10% sensitization for populations with diagnosed allergic diseases and more than 3% for other cohorts were included based on the difference in sensitization rates in atopic and general populations [30,31,35,36,37]. For populations with severe allergic diseases and very high sensitization rates, only the leading sensitizers were indicated. For grouping studies in synthesis, publications were sorted by region and publication date, from the earliest to the most recent (Section 3.1). As measures of heterogeneity, numbers and characteristics of study populations as well as diagnostic methods determining the sensitization were taken into account.

### 2.4. Assessment of Methodological Quality and Certainty of Evidence

The methodological quality of each study was evaluated using the Joanna Briggs Institute (JBI) Critical Appraisal Checklists. Certainty of evidence was assessed using the Grading of Recommendations Assessment, Development and Evaluation (GRADE) framework rating each study across the domains of risk of bias, inconsistency, indirectness, imprecision, and publication bias. The interpretation of indirectness was adapted to the context of sensitization profiling. In particular, we did not automatically privilege extract-based SPT or sIgE as the “standard” method; instead, we considered the appropriateness and validity of the diagnostic method for the accurate IgE sensitization determination. This allowed us to avoid penalizing studies that used more advanced and mechanistically accurate molecular diagnostics. Risk of bias was independently assessed by the primary reviewer with the assistance of an AI-based analytical tool to minimize subjective bias; in cases where the two assessments differed, a second reviewer was consulted to reach consensus.

### 2.5. Subgroup and Sensitivity Analysis

Numeric sensitization percentages were extracted. For each relevant allergen (birch, grass, weed, house dust mite (HDM), cat, peanut, cow’s milk, egg), only explicitly reported overall percentages were included; studies reporting only subgroup strata (age strata) without a single overall percentage were excluded for that allergen. For studies reporting ranges without a single value, the midpoint was used only when no single value was provided. Each study was classified by modality (SPT only; extract only serology; molecular/mixed; non-conventional experimental panel) and by panel completeness (Full = pollen + indoor + food present; Restricted = missing one or more groups). We computed summary statistics per allergen and repeated these summaries stratified by modality, panel completeness and year groups where data permitted. A sensitivity analysis excluded non-conventional platforms (RIDA AllergyScreen^®^, Polycheck^®^, Protia Allergy-Q^®^, hydrogel chips, custom ELISA) and compared medians before and after exclusion. Single study strata are reported but flagged as low evidence.

### 2.6. Visualization

The images were created using Adobe Illustrator (2025 29.8.3.3 x64) (Adobe Inc., San Jose, CA, USA), Microsoft PowerPoint (2016 16.0.4266.1001 32-bit) (Microsoft, Redmond, WA, USA), and GraphPad Prism 10.0 (Dotmatics, Boston, MA, USA). The geographic information system QGIS (3.34) (QGIS Development Team, QGIS Geographic Information System, Open Source Geospatial Foundation Project) was used to create a population density map. The cartographic basis was acquired as digital vector data of the administrative–territorial division of Russia. Statistical information on population density was obtained from the official data of the Federal State Statistics Service (Rosstat). Thematic mapping methods using color gradation were applied to visualize population density. JBI checklist evaluation and GRADE assessment were visualized using Robvis and Critiplot online tools, respectively [38,39].

## 3. Results

### 3.1. Regional Sensitization Patterns and Allergen Panel Coverage

**Figure 3:**
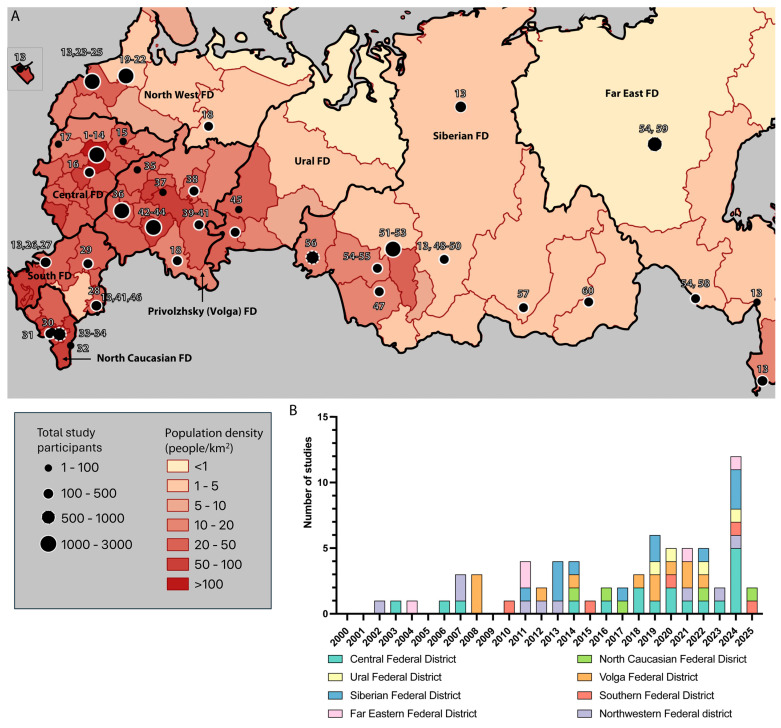
Distribution of available sensitization profiles across location and time. (**A**) Geographic distribution of the studies summarized in Table 1. The numbers on the map are consistent with the number of studies in the column “No.” of Table 1. Black circles indicate the total number of participants at each location; the color scale represents population density. (**B**) Number of studies conducted in each federal district over time. Colored bars correspond to the following federal districts: Central (turquoise), Ural (yellow), Siberian (blue), Far Eastern (pink), North Caucasian (light green), Volga (orange), Southern (red), and Northwestern (purple). The *y*-axis shows the total number of studies conducted per year.

Data were collected and analyzed from eight federal districts (FD) of the Russian Federation (Central, Northwestern, Southern, North Caucasian, Volga, Ural, Siberian, and Far Eastern FD) and 36 constituent entities of the Russian Federation, respectively (Table 1). Figure 3 depicts the geographical location of the studies, the number of participants per region (Figure 3A), and the distribution of the data publications over time (Figure 3B). Out of 60 eligible studies, 9 (15%) analyzed samples from the eastern half of the country, which is proportional to the population density being the highest in the south and western regions (Figure 3A). Almost two thirds of the studies (38/60) were published within the last decade, suggesting that the available evidence is relevant (Figure 3B).

Considering the allergen spectrum to be covered when pollen (birch, grass and weed), indoor (animal, HDM and fungi) and food allergens are included in the assessment, only 12 studies (20%) could be classified as describing the full profiling, whereas others lacked one or more groups of allergens in their testing strategy. Seven studies (11.7%) used allergen molecules to dissect the sensitization pattern, while the majority relied on extract-based diagnostics (Table 1).

Six studies reported the usage of internationally validated inclusion criteria such as the International Study of Asthma and Allergies in Childhood (ISAAC)-based questionnaire [45,59,73,75,86,99]. None of the profiling studies considered whether participants were long-term residents of the region, which limits the ability to link sensitization patterns to the exposome peculiarities of the given region. The absence of AIT history was taken as an enrolment prerequisite in two studies [43,45]. Five studies included both patients with allergy symptoms and a control group or participants without allergy symptoms [45,78,89,92,94]. Venom allergens provoking serious anaphylactic reactions, as well as certain fungi (e.g., *Malassezia*), were absent from all diagnostic panels besides micro- and macro-arrays (e.g., ImmunoCAP^®^ ISAC, ALEX2^®^, MeDALL).

In the Central FD, 14 out of 17 studies were conducted in Moscow and the Moscow region (No. 1–14 in Table 1 and Figure 3). The sensitization pattern was characterized by prominent tree pollen sensitizations dominated by birch and cross-reactive tree pollens, and animal allergens (cat and dog) [40,41,43,45,46,49,50,51,52,54,55,56]. Studies from adjacent regions of Smolensk and Tula (No. 17 and 16 in Table 1 and Figure 3, respectively) reported broadly similar patterns, although differences in panel composition limit direct comparison [55,56]. Several cohorts in the Central FD have shown high HDM sensitization prevalence [40,54,56]. Studies focusing on food sensitizations revealed moderate sensitization to cow’s milk and chicken egg; the data with high food sensitization rates were mainly obtained from pediatric cohorts [40,43,44,47].

Northwestern FD was characterized by nine studies that revealed high significance of HDM and cat allergen sensitizations [52,57,58,59,60,61,62,63,64]. Birch and grass pollen sensitizations were observed often but the extent varied across studies, reflecting methodological heterogeneity. In contrast, sensitization profiles of Southern FD described in five publications have shown strongly prevailing weed sensitization with regional differences: ragweed was most notable for the Rostov (No. 26–27 in Table 1 and Figure 3) region, goosefoot for Astrakhan (No. 28 in Table 1 and Figure 3), and mugwort for Volgograd (No. 29 in Table 1 and Figure 3) [52,65,66,67,68]. Grass pollen sensitization was also reported, although the data remain limited by incomplete panel coverage in southern regions.

Likewise, North Caucasian FD populations analyzed in five studies were largely sensitized to weeds but also to HDM and grasses [69,70,71,72,73]. Volga FD is characterized by the largest number of constituent entities covered (seven regions) and with tree pollen being a major sensitization source, however the evidence was heterogeneous, and many studies lacked full allergen coverage (e.g., they used only pollen allergens) [57,74,75,76,77,78,79,80,81,82,83]. Tree pollen and cat allergens were noted to be the main sensitizers in Ural FD [52,80,84,85].

In Siberian FD, 12 studies were performed in 7 constitutive entities, however 6 of them were narrowly focused on food or fungi allergens thus not allowing wide profiling [87,88,89,90,91,94]. Notable allergen sources here were cat, HDM, birch pollen, chicken egg and cow’s milk [52,92,93,95,96]. Far Eastern FD was characterized by prominent HDM reactivity followed by animal allergen sensitization [52,93,97,98,99]. These two FDs are the largest constituent entities where the study centers are very distant.

Across studies, tree pollen (particularly birch) and animal allergens (especially cat) were frequently reported sensitizers. Weed sensitization was commonly reported in southern regions, although the extent is difficult to quantify due to methodological variability. Grass pollen and HDM were characterized by notable sensitization rates in several centers; however, interregional differences are not reliably identifiable due to the heterogeneity of the methodology and study populations. Harmonized methodology will be essential for future sensitization mapping studies in order to obtain reliable data on IgE recognition profiles.

### 3.2. Risk of Bias and Certainty of Evidence

Risk of bias was assessed using the JBI checklist for prevalence studies. Across the 60 included studies, the overall risk of bias was predominantly high. Only several large, population based studies were judged to have low overall risk of bias [58,59,60,61]. Seven further studies were judged to have some concerns [42,43,45,46,49,53,92], while the remaining 49 studies (81.7%) were rated as having a high risk of bias (Figure S1). The main drivers of a high risk of bias were an inappropriate sample frame and sampling method. Most studies recruited patients from allergy, pulmonology, or dermatology clinics. Coverage of the overall population was therefore judged inadequate in almost all clinic based studies. Reporting of response rates was uniformly poor: the response rate domain was rated as “No information” in nearly all studies, including those with otherwise acceptable methods. Subjects and setting were usually described only partially, with limited information on recruitment pathways. Measurement domains were generally acceptable. Most studies used standardized SPT extracts and/or validated in vitro sIgE assays (ImmunoCAP^®^, ImmunoCAP^®^ ISAC, ALEX2^®^, MeDALL), and applied the same panel to all participants. As a result, the “Valid measurement” and “Same measurement for all” domains were typically rated as low or at most some concerns. However, these strengths did not compensate for the structural weaknesses in sampling and reporting, and the overall risk of bias is driven by sampling frame, recruitment, and reporting, not by the diagnostic platform.

Certainty of evidence was assessed using GRADE, focusing on risk of bias, inconsistency, indirectness, imprecision, and publication bias (Figure S2). Overall, the certainty of evidence was low to very low for the vast majority of studies. Risk of bias was rated as serious in 93% of studies, reflecting the JBI findings. Indirectness was a major and systematic limitation. Almost all studies were clinic-based and recruited only symptomatic patients with only certain disease entities, without control group enrollment, making them indirect for estimating IgE reactivity profiles and sensitization prevalence in the general population. These studies were therefore downgraded for serious or very serious indirectness. Only the population-based cohorts and the EuroPrevall study were judged to have not-serious indirectness [58,59,60,61,92]. Imprecision was frequently serious or very serious, particularly in small or highly selected clinic samples, in studies with small tested subsets, and in those that did not report confidence intervals. Imprecision was judged not serious only in the large population-based cohorts and a small number of larger, well-powered laboratory cohorts. Inconsistency was generally not serious: despite methodological limitations, patterns of sensitization across studies were broadly similar, with no strong evidence of conflicting results. Publication bias was considered generally not serious due to the near absence of null or negative reports except for several studies reporting the results only partially. Thus, GRADE overall certainty was moderate in 4 studies (6.7%), low in 2 studies (3.3%), and very low in the remaining 54 studies (90%) (Figure S2).

Importantly, more sophisticated laboratory methods (e.g., ImmunoCAP^®^, ISAC, ALEX2^®^) did not systematically translate into higher certainty of evidence. Molecular and multiplex platforms improved the “Valid measurement” domain but did not overcome the fundamental limitations of clinic-based sampling, unclear sampling frames, and small or selected samples. The only studies that achieved moderate certainty were those with population-based sampling frames and robust epidemiologic design, all of which used conventional extract-based SPT and/or ImmunoCAP^®^.

### 3.3. Assessment of IgE Sensitization Diagnostic Modalities

The diagnostic modalities used across studies differed substantially in their inherent measurement biases, and this heterogeneity directly affects the interpretation of sensitization prevalence (Table 2). SPT, used in 24 studies (40%), may overestimate sensitization because extract-based reagents contain multiple cross-reactive proteins and because SPT detects mast-cell-bound IgE, which may yield positive reactions even when circulating IgE is below the detection limit or absent. Extract-based serology (9 studies, 15%) may lead to higher apparent sensitization rates, as extract mixtures still include cross-reactive components, yet serology is less sensitive than SPT and does not detect tissue-bound IgE. In contrast, single-allergen molecular assays (3 studies, 5%) such as ImmunoCAP^®^ components, ISAC, or MeDALL provide higher specificity, removing much of the cross-reactive background and identifying true primary sensitizers, though they may appear less sensitive when compared with broad extract reactivity.

Mixed extract-plus-molecular approaches (11 studies, 18%) yield a balanced profile, provided that the screening panel is sufficiently comprehensive; however, this was not always the case in the present study selection, meaning that the balance between extract-driven inflation and component-driven specificity varied across studies. Finally, experimental assays (13 studies, 22%), including custom ELISAs, hydrogel biochips, RIDA Allergy-Screen^®^, Polycheck^®^, and Protia Allergy-Q^®^, show variable performance and may introduce bias due to limited standardization and potential nonspecific binding or insufficient sensitivity, and therefore require cautious interpretation.

### 3.4. Subgroup and Sensitivity Analysis of Allergen Sensitization Prevalences in Moscow Region

Subgroup and sensitivity analyses were performed only for Moscow and Moscow-region studies because these constituent entities contributed the largest number of methodologically diverse datasets, allowing meaningful stratification by diagnostic modality, panel completeness, and age structure (Table S1). Birch pollen sensitization was frequently reported to be high across pediatric and adult cohorts. Grass pollen sensitization was reported in multiple pediatric and mixed cohorts; the sensitization levels were moderate but heterogeneous. A prominent sensitization to weed pollen was reported in a single study evidence [41]; it must be noted that molecular arrays include weed allergens but only one study in this subset reported a sensitization rate >10%. Cat sensitization was reported in both pediatric and adult populations; molecular/mixed study reports the highest cat component frequencies. HDM values vary by panel and platform; this heterogeneity is driven by one experimental panel and one full panel SPT study. Milk and egg sensitization was reported mainly from pediatric cohorts and no full panels have indicated them as top sensitizers. Peanut numbers come mainly from pediatric or pediatric-focused cohorts; however, in this analysis, no distinction was made between class I allergens, which are more often genuine sensitizers, and highly cross-reactive class II food allergens.

Within this subset, substantial variation in sensitization prevalence was attributable to methodological differences: exclusion of experimental platforms altered medians for several allergens, most notably decreasing HDM and increasing cat, peanut and egg sensitization. Age composition further shaped the results, with food sensitizations reported predominantly in pediatric cohorts and molecular adult cohorts showing higher birch component frequencies. No temporal trend could be inferred due to confounding by methodological differences.

## 4. Discussion

### 4.1. Outcomes of the Existing Data and Its Limitations

We have collected and summarized the available evidence on allergic sensitization profiling in Russia published to date. The systematic search yielded 60 studies fulfilling the inclusion criteria. The chosen thresholds for relevant sensitization rates are based on previous reports of microarray profiling studies: for example, Kazancioglu et al. have shown the ranges of 12–57% for symptomatic and 4–21% for asymptomatic subjects, and in the work of Kiewiet et al. on cohort studies, most frequently recognized allergens ranged between 16 and 30% sensitization; thus, these thresholds (>10% for symptomatic and >3% for population/asymptomatic cohorts) are intended to capture the most frequently reported sensitizers across studies with differing diagnostic sensitivity [30,31,35,36,37]. A distinct variety of the relevant allergens across different regions have been observed resulting from diverse climatogeographical and behavioral patterns. Indoor and animal allergens were shown to be important in northern regions whereas sensitizations to weeds such as mugwort and ragweed were prevalent in the southern regions. Tree pollen, particularly birch, as well cat allergens were shown to be frequently reported sources of IgE sensitization across multiple studies highlighting the importance of developing specific therapeutic as well as prophylactic measures targeting these respiratory allergens.

There were several limitations of this study that we would like to point out.

(1) *Sampling and recruitment limitations.* Varying availability of specialized medical care across different regions may affect the detection rate of allergic diseases in the region overall, as well as the ability to recruit patients from different parts of the region for participation in specific studies, which influences the completeness of population coverage and data representativeness in each individual study.

(2) *Cohort heterogeneity.* Small sample sizes in some studies, and insufficiently balanced representation of different age groups and health conditions, as well as the absence of uniform standards for data collection, analysis, and interpretation among studies within the same region complicate the interpretation of results and limit the ability to draw conclusions for a given region as well as to compare the data from different constituent entities. Inclusion and exclusion criteria play a key role in the study outcome, and this factor was highly heterogeneous in the studies analyzed. For example, in the study by Mokronosova et al., only patients with a history of reactions to two or more allergens were included, and only Phadiatop-positive patients were enrolled, hence this population comprises patients with complex atopy [48]. The general prerequisite is that most of the studies were conducted in specialized allergology clinics, where mostly patients with moderate to severe diseases end up. The sensitization prevalence and magnitude of reactions varied according to cohort characteristics, underscoring the need for more standardized recruitment strategies in future profiling studies.

(3) *Diagnostic modality heterogeneity.* The major drawback is the diversity of methods assessing the presence of allergic sensitization. The publications included in the review employed various methods such as skin testing and specific IgE determination using various systems (ImmunoCAP^®^, ImmunoCAP^®^ ISAC, ALEX2^®^, MeDALL, Polycheck^®^, hydrogel biochips, Protia Allergy-Q^®^, UniCAP^®^). The diversity of diagnostic approaches and the fundamental difference in information derived from using allergen extracts versus allergen molecules makes it difficult to compare data and interpret results across studies. For sIgE determination, the results differ substantially depending on the detection method, even when produced by the same company, and highly standardized and clinically validated methods, e.g., ImmunoCAP^®^ and ImmunoCAP^®^ ISAC, show notable differences in the measured outcomes [100]. Allergen extracts, used for both serological and skin testing, contain not only genuine source-specific allergens, but also cross-reacting compounds. On the contrary, molecular allergy diagnostics, or component-resolved diagnostics (CRD), can provide additional resolution by identifying the culprit allergens at the molecular level and thus differentiate genuine sensitization from cross-reactivity to homologous molecules [101]. Additional benefits of CRD include assessment of disease severity and determination of AIT strategy [102]. However, in our selection, only seven studies used molecular testing, and the existing data are predominantly extract-based.

Different correlations of sIgE determination and skin testing results were observed: from only 59% [65] to almost identical results by both methods [73]. Several studies reported large discrepancies between skin testing results and measurements of sIgE [58,59,78,88]. This might be attributed to several reasons, such as the difference in compounds for skin and laboratory testing, the use of medications, or the skin’s reactivity and condition; sIgE assays may also miss low-level sensitizations, which nevertheless can produce a skin reaction. The presence of cross-reactive carbohydrate determinants (CCDs) in natural and eukaryotic cell-expressed recombinant allergens may result in positive sIgE results without clinical relevance, whereas recombinant allergens produced in prokaryotic expression systems do not show such reactivity [103,104]. Our findings indicate that these modality-dependent mechanisms are likely a major source of the inconsistent sensitization profiles observed across studies: SPT may inflate prevalence through mast-cell-bound IgE and cross-reactive extract material, extract-based serology produces a moderately inflated but narrower signal, and molecular assays reveal a more specific set of true primary sensitizers. Mixed extract-plus-molecular approaches only yield a balanced picture when their panels are comprehensive, which was largely not the case in our dataset. Thus, ideally, both skin testing and sIgE determination should be present in a profiling study to improve interpretability.

(4) *Narrow allergen panels.* In a number of publications, the primary focus was only a narrow selection of allergens (plant pollen, household, animal or food allergens). The selection in this case likely was driven by pre-existing empirical knowledge of relevant allergen sources, or a specific focus of the research question in the particular publication. The inclusion of patients with only positive sIgE or skin testing creates the enhanced recognition frequency of known allergens and neglects the possible presence of sensitizations to the potentially relevant allergens yet absent in the diagnostic panels. Less-studied allergens could provide additional valuable data for understanding sensitization profiles in different regions; thus an appropriate profiling study should include the widest possible range of allergens.

(5) *Temporal trends in sensitization patterns could not be reliably traced.* The combination of heterogeneous methods, changing panel breadth, differing age structures, and varying inclusion criteria across roughly two decades of studies prevents any robust quantification of change over time. Birch and cat emerge as dominant sensitizers in central and northern regions in both older and more recent work, but the available data do not allow conclusions about temporal change. Weed sensitization (ragweed, goosefoot, mugwort) appears strongly in more recent Southern and North Caucasian studies that actually include these allergens, but earlier absence is best interpreted as panel omission rather than evidence of low prevalence. For food allergens such as cow’s milk, egg or peanut, the evidence is fragmented, often pediatric only and methodologically inconsistent, so no defensible temporal trend can be inferred. Any mention of “increase” or “rise” over time would therefore be confounded by evolving diagnostic strategies and should be explicitly framed as non-interpretable in temporal terms.

(6) *High risk of bias and limited quantitative assessment.* The formal risk of bias assessment underscores that unsystematic study design and incomplete methodological transparency remain major obstacles to generating reliable sensitization profiles. Restricting subgroup and sensitivity analyses to the Moscow/Moscow region studies was necessary because only this region provided enough studies with heterogeneous methods, panel structures and age distributions to support valid stratified comparisons. In other federal districts, most allergens were reported by one or two studies only, making subgroup medians mathematically equivalent to single study values and sensitivity analyses uninformative.

### 4.2. Characteristics of the Prototype Studies Suitable for IgE Sensitization Profiling

The Moscow subset illustrates how strongly diagnostic modality, panel completeness and age composition influence reported prevalence, underscoring that interregional or temporal comparisons cannot be interpreted without controlling for these factors. These findings highlight the need for harmonized, region-independent diagnostic strategies if future sensitization mapping is to yield interpretable temporal or geographic patterns.

One single-center cross-sectional study of the IgE sensitization profile was exemplarily performed in the pediatric population of the Moscow and Moscow region: two groups (*n*~100 in each) of age- and gender-matched children with and without allergy symptoms according to the international ISAAC questionnaire; with sIgE profiling assessed by microarray containing > 160 allergen molecules and accompanying skin testing with a panel of aeroallergen extracts [45]. The only drawback was that no information on birthplace and/or duration of residency in the region was collected, however for children, the likelihood of a migration background, and thus the sensitization profile not matching the current place of residence, is minimal. Thus, such work could be considered as a prototype of future studies on IgE sensitization profiling of the country for more centers. The profiling study of Borisov et al. features good coverage of the country and systematic sample collection; however, in comparison with the aforementioned work of Elisyutina et al., the inclusion criteria were not internationally validated, no asymptomatic children were included for comparison, and the allergen panel is extract-based and rather small. The major disadvantage is the method of data evaluation: the most frequent sensitizers were reported for the whole study population only, which is not very informative given the large and diverse territory of the country; and for the study centers separately, the sensitizations are only presented as % among household/pollen/food sensitizers, but the frequencies of sensitizations for different centers are not reported [52].

Thus, studies with a comprehensive, consistent and reproducible methodology for participant recruitment and subsequent data acquisition and analysis would provide a foundation for future region-tailored approaches and allergy prevention strategies. A prototype study capable of generating such evidence would require harmonized, age-stratified recruitment of both symptomatic and asymptomatic individuals directly from the general population in regional urban centers, where outpatient facilities can realistically support standardized enrollment procedures. Classification of participants should rely on an internationally validated questionnaire, e.g., ISAAC-based, ensuring consistent symptom definitions across sites. To link IgE reactivity patterns to the local exposome, residence in the region for a predefined minimum period must be an inclusion prerequisite. Because clinical relevance of IgE sensitization is most reliably captured once sensitization has reached its full phenotypic expression, initial data collection in adult populations would provide a clear representation of established sensitization profiles. All enrolled participants should undergo a uniform diagnostic work-up consisting of complete SPT extract panels and sample collection for molecular sIgE profiling using a microarray, with all laboratory analyses ideally centralized in a single reference facility to ensure methodological consistency. Such sensitization studies have not yet been published for Russia and remain uncommon internationally, thus the study design outlined here is intended as a universally applicable model for future work in this area. Together, such a design would yield robust, region-specific sensitization maps suitable for informing the subsequent development of allergy management strategies.

### 4.3. Considerations of IgE Sensitization and Its Dynamics in the Context of Clinical Relevance

A systematic collection of data on IgE sensitization across different populations is essential for interpreting regional IgE reactivity patterns in a clinically meaningful way. Respiratory allergens warrant particular attention: they are highly prevalent and, unlike many food allergies, respiratory allergies tend to persist or progress across the lifespan. In a longitudinal study from Karelia—a 10-year follow-up of a subset (*n* = 82) of the original study population (*n* = 427)—the prevalence of sIgE to inhalant allergens nearly doubled, whereas IgE recognition of food allergens (milk and egg) decreased by approximately half [59,105]. This pattern is consistent with the characteristic trajectory of the atopic march, which often begins with food-related reactions in early childhood and progresses toward sensitization to a handful of respiratory allergens later in life [35,105].

On the individual level, the established sensitization profiles are considered to stay consistent without new sensitizations appearing in adulthood [9]. However, local sensitization profiles may shift along with a generation change due to environmental and habitual alterations. The work by Akhapkina et al. clearly illustrates such changes in the obtained sensitization frequencies in methodologically similar studies performed in the same center with a distance of 10 years: pollen sensitization rate became 15% more frequent, whereas sensitization to HDM dropped by almost two times [51]. In another study, similar findings alongside an increase in cat allergen sensitization from 18 to 64% were observed [63]. Another method to study the shift in sensitization profiles is the cross-sectional assessment of different generations where notable changes were also observed [75,106]. Another long-term shift was described for the Rostov region, where the SPT positivity to ragweed increased drastically from 13.4% in 1972 to 82.1% in 2014 in the seasonal allergic rhinitis population [65]. Hence, once established, the “sensitization map” in the given region may still evolve over time and require periodic reassessment.

A frequently detectable IgE binding to the allergen does not necessarily indicate clinical relevance. For example, many individuals having venom-specific IgE never experience an inadequate sting reaction [107,108]. Highly cross-reactive plant allergens such as profilins and polcalcins exhibit moderate to low clinical relevance, and CCDs exhibit low to no relevance [104,109,110]. Albumins are also highly cross-reactive, often asymptomatic but nevertheless clinically relevant in certain contexts [111]. Detection of sIgE to such allergens often does not require AIT prescription. In contrast, some molecules, such as Bet v 1, Phl p 1, Phl p 5, and Fel d 1, are strongly associated with symptomatic disease, and the detection of specific IgE to them suggests considering AIT to prevent disease progression [112]. Thus, differential consideration for the detected sensitization should be given depending on the pathogenic potential of the identified IgE-binding allergens, and therefore studies based solely on the use of extracts might not reliably identify clinically relevant sensitizations.

A growing body of evidence shows that many population-level molecular sensitization patterns can be mechanistically explained by the properties of allergens and the immunological pathways they activate. Primary sensitizers tend to be structurally stable proteins capable of surviving environmental or mucosal barriers, such as Der p 1/Der p 2 with intrinsic protease activity that disrupts epithelial junctions and promotes Th2 priming, or Bet v 1-like PR-10 proteins whose labile structure leads to strong IgE responses but limited systemic reactivity. Age-dependent immune ontogeny further shapes these profiles: infants preferentially mount Th2-skewed responses to epithelial-disruptive allergens, whereas tolerance is more likely when exposure involves non-proteolytic molecules. Together, these mechanisms suggest that regional and age-specific sensitization patterns arise from predictable interactions between allergen structure, exposure biology, and immune system development. Recognizing these mechanistic underpinnings helps to interpret the sensitization patterns observed in our dataset and provides context for discussing how such profiles can be used in future allergy research.

## 5. Conclusions

Here, we present the characteristics of the IgE sensitization profiles in Russia available to date, identify the existing knowledge gaps, and outline the prototype of studies needed to address them, as well as the regional allergen-related research questions that could be explored in future on the basis of such data. We consider it important to demonstrate why, despite a seemingly large evidence base that already includes modern molecular diagnostic methods, it remains impossible in this case to construct a robust, population-level sensitization mapping in a country with diverse environmental landscapes. The problem is not the absence of data but the absence of systematic, method-aware, and regionally coherent evidence. Addressing this gap will depend on generating harmonized sensitization profiles across regions, which can provide the empirical basis for future discussions about how IgE sensitization data might be incorporated into broader approaches to allergy management.

## Figures and Tables

**Table 1:** Studies included in the final synthesis.

Region	No.	N (m) *	Age (Aver.) **	Population	Testing Methods	Sensitizations	Comments, Limitations	Citation
**Central Federal District**								
Moscow and Moscow region	1	20 (60)	1–4 (3)	allergic, born to parents with hay fever	Polycheck^®^, Milenia Biotec: sIgE to peanut, milk, egg white and yolk, potato, carrot, codfish, apple, soya, wheat; birch, timothy grass and ragweed pollen; *D. pter.*, *D. far.*; dog, cat and horse epithelium; *A. fumigatus*, *C. herbarum*	65% *D. pter.*, 35% dog epithelium, 30% birch, 25% cat, 25% peanut, 20% apple, 15% milk, 15% egg white, 10% timothy grass pollen, 10% codfish, 10% *D. far.*	20 components Extract-based No mugwort	Mokronosova et al. 2003 [40]
	2	195	3–52	allergic	Skin testing to tree (birch, alder, hazel), grass (cock’s foot), weed (mugwort) pollen and fungi (*A. alternata*, *C. herbarum*, *R. nigricans*, *P. notatum*, *C. albicans*, *A. fumigatus*) allergens	76.14% birch, 69.32% alder, 57.95% hazel, 39.77% cock’s foot, 55.68% mugwort and 56.82% *A. alternata*	11 components Extract-based No animal, HDM and food allergens	Akhapkina et al. 2014 [41]
	3	682 (55)	2 m–17	AD, FA	ImmunoCAP^®^, Phadia: sIgE to allergens of cereals (wheat, rye, oats, barley, corn, rice, buckwheat), vegetables (potatoes, carrots, cabbage, broccoli, pumpkin, beets, tomatoes), bananas, meat and poultry (beef, pork, rabbit, lamb, chicken, turkey)	To plant allergens: 2–5 m—potatoes, pork and cereals (8–14%); 6–18 m—potatoes (22.7%), buckwheat (19.3%), cereals (10−15%); 1.5−4 y—bananas (29.5%), cereals, carrots and potatoes (19.5–24%); 4–10 y—bananas, carrots and cereals (20–28%); 10–17 y—carrots (47.5%), bananas, cereals and tomatoes (30–36%). Meat or poultry specific IgE were observed in 8–15% of cases	21 components Extract-based Food allergens only	Snovskaya et al. 2016 [42]
	4	51 (56)	6 m–17	AD, AR, BA	Hydrogel biochip: sIgE to tree (birch, alder, hazelnut, oak), weed (wormwood, mugwort, dandelion), grass (bermuda grass, cock’s foot, meadow fescue, perennial ryegrass, rye) pollen, indoor (cat and dog dander, *D. pter.*, *D. far.*, cockroach), fungal (*A. tenius*) and food (egg white, cow milk, α-lactalbumin, β-lactoglobulin, casein, codfish, wheat, peanut, hazelnut, carrot, apple, peach) allergens	Most prominent to birch (32%) among pollen allergens and cat dander (24%) among indoor allergens, these sources have also shown age-related elevations. IgE to egg whites (21%) was the most prevalent among food allergens	31 components Extract-based No ragweed	Voloshin et al. 2018 [43]
	5	129 (52)/81 (53)	3 m–17	vegetarian/control	ImmunoCAP^®^, Phadia: sIgE to allergens of cow’s milk, soy, beef, pork, chicken, codfish, egg white and wheat	Vegetarian/control groups: 12.4/12.3% cow’s milk, 10.1/8.6% egg white, 4.6/4.9% wheat and 5.4/0% soy. Food allergy-related symptoms were registered in 65.1% of cases with 51.1% as skin reactions	8 components Extract-based Food allergens only	Yasakov et al. 2019 [44]
	6	103 (56)/97 (55)	median (SD) 12 (2)/13 (2)	allergic/control	sIgE to micro-arrayed allergen molecules (MeDALL) + skin testing	Aeroallergens: dominated by Bet v 1 (allergic: 63.1%; control: 25.7%) and Fel d 1 (allergic: 61.1%; control: 15.4%), in symptomatic children followed by Phl p 1 (27.1%), Can f 1 (26.2%), Cup a 1 (18.4%), Art v 1 (16.5%), Pla a 2 (15.5%), Cry j 1 (13.5%), Amb a 1 (12.6%) and Der p 2 (19.7%) with similar pattern but lower frequencies in control group. Food allergens reactivity was observed mainly to cross-reactive PR-10 proteins	160+ components Molecular-based ISAAC questionnaire	Elisyutina et al. 2020 [45]
	7	153 (58)	1–17 (9 ± 3)	allergic	ImmunoCAP^®^, Phadia: sIgE to 28 allergens of tree, grass, weed pollen, animal allergens, dust, food allergens	Most prominent to alder (85%) among tree pollen, timothy grass and meadow-grass (70%) among grass pollen and mugwort (53%) among weeds; 22% to house dust, 19% to cat and 13% to dog	28 components Extract-based No fungal allergens	Levina et al. 2021 [46]
	8	79	2 m–24 m (13 ± 7 m)	AD	Skin testing to allergens of egg, cow’s milk, fish, wheat, nuts and soy	38.0% egg, 27.8% cow’s milk, 10.1% fish. At 2–6 m sensitization was found in 36.4%, and after 6 m in 57.9%. No differences between exclusively breastfed and formula-fed children were observed	6 components Extract-based Food allergens only	Smolkin et al. 2022 [47]
	9	163 (56)	7–48	AD, AR, atopic BA, FA	ALEX2^®^, MacroArrayDX: sIgE to 178 allergen molecules and 117 allergen extracts	To storage proteins (12 2S albumins, 4 7/8S globulins and 4 11S globulins) detected in 21.5%, most frequently to 7/8S globulins Jug r 2 (11%) and Cor a 11 (10%)	295 components, data reported for 20 only Mixed	Mokronosova et al. 2023 [48]
	10	556 (46)	6–58	allergic	ALEX2^®^, MacroArrayDX: sIgE to 178 allergen molecules and 117 allergen extracts	Sensitization to Bet v 1 was observed in 42% of cases. Sensitization rate to “panallergens” (6 profilins, 2 polcalcins, 12 nsLTP, 7 parvalbumins) was less than 10%	295 components, data reported for 28 only Mixed	Zheltikova et al. 2024 [49]
	11	100 (42)	adults (44 ± 14)	severe AD and BA	ImmunoCAP^®^ ISAC, Phadia: sIgE	Bet v 1 (60.3–90.0%) and cross-reacting PR-10 proteins; group 1 grass pollen allergens Phl p 1 (41.3–75.0%) and Cyn d 1 (19.0–55%); lipocalins Can f 1 (36.5–88.2%), Can f 4 (15.0–58.8%), Can f 6 (25.0–64.7%) and Ecu c 1 (22.2–64.7%) as well as animal allergens Can f 5 (31.7–64.7%), Fel d 2 (15.0–47.1%) were the most frequent sensitizers among all groups	112 components Molecular-based	Fomina et al. 2024 [50]
	12	72 (69)	4–62	allergic	Skin testing to tree (birch, alder, hazel), grass (timothy grass, cock’s foot) pollen and HDM (*D. pter.*, *D. far.*) allergens	80.56% birch, 72.22% hazel, 70.83% alder, 44.4% timothy grass, 43.06% cock’s foot, 31.94% *D. pter.*, 29.17% *D. far.*	7 components Extract-based No animal, fungi, weed and food allergens included	Akhapkina et al. 2024 [51]
	13	51 (53)	3–12 (7)	allergic	Protia Allergy-Q^®^, ProteomeTech: sIgE by enzyme immunoassay	Cat was the most prominent (40% of indoor allergens sensitizations) in indoor allergens, birch—in pollen (27.58% of pollen sensitizations) and none of the food allergens was clearly dominating	43 components Extract-based	Borisov et al. 2024 [52]
	14	240 (60)	0–17 (median 8)	AD, AR, FA	sIgE tested by: ImmunoCAP^®^, Phadia (milk, wheat, codfish, egg white); ImmunoCAP^®^ ISAC, Phadia; ALEX2^®^, MacroArrayDX	PR-10 proteins (57% hazelnut, 47% peanuts, 39% soy, 24% walnut), chicken egg—18%, cow’s milk—12%. Only 30.8% had symptomatic sensitization to food allergen, of those 13% to hazelnut	295 components, data reported only for food allergens Mixed	Efendieva et al. 2024 [53]
Yaroslavl region	15	41/38	18–60/ 3–17	allergic: adults/children	Skin testing to tree pollen (birch, mix), grass mix and weed mix, fungi (*A. alternata*, *C. herbarum*, *P. notatum*, *A. fumigatus*), HDM (*D. pter.*, *D. far.*) and animal (cat and dog dander) allergens	HDM 25.0/26.2%, tree pollen 26.0/23.1%, animal dander 16.7/20.0%, grass pollen 15.6/7.7%, weed pollen 10.4/13.9%	10 components Extract-based No food allergens	Vorontsova et al. 2018 [54]
Tula region	16	103	5–14	AR	Skin testing to tree (birch, alder), grass (timothy grass, meadow fescue, cock’s foot, ryegrass) and weed (mugwort, sunflower) pollen	50.7% timothy grass, 48.0% cock’s foot, 48.0% birch, 45.4% ryegrass, 42.7% meadow fescue, 30.7% sunflower, 25.3% mugwort, 22.7% alder	8 components Extract-based Pollen only, no ragweed	Bergets et al. 2007 [55]
Smolensk region	17	45 (84)	4–8	AR	Skin testing to tree, grass and weed pollen and HDM allergens	HDM 64.4%, tree pollen 53.3%, grass pollen 46.7%, weed pollen 37.8%	4 components Extract-based No animal, fungi and food allergens	Bekezin et al. 2020 [56]
**Northwestern Federal District**								
Kaliningrad region	13	55 (29)	3–9 (4)	allergic	Protia Allergy-Q^®^, ProteomeTech: sIgE by enzyme immunoassay	Cat was the most prominent source among indoor allergens (42.76% of indoor allergens sensitizations), birch—in pollen allergens (40% of pollen sensitizations) and egg—in food allergens (34.78% of food sensitizations)	43 components Extract-based	Borisov et al. 2024 [52]
Komi Republic	18	293	14–70	allergic	Skin testing to tree (birch, alder, poplar, hazel, pine, spruce, lilac), grass (timothy, cock’s foot, fescue, brome, meadow-grass, meadow foxtail, rye, perennial ryegrass, couch grass, corn), weed (mugwort, nettles, goosefoot, plantain, dandelion, sunflower, ragweed) pollen allergens	Most prominent to birch (54.6%) among tree pollen allergens, timothy grass (48.8%) among grass pollen allergens and mugwort (34.5%) among weed pollen allergens	24 components Extract-based Pollen allergens only	Vahnina et al. 2012 [57]
Republic of Karelia	19	385	25–54	random population	Skin testing (birch, timothy grass, mugwort, cat, dog, horse, cow, *A. alternata*, *C. herbarum*, *D. pter.*, cockroach), sIgE by Pharmacia CAP (birch, timothy, *C. herbarum*, *D. pter.*, cat)	11.7% *D. pter.*, 3.9% timothy grass as defined by skin testing and 9.2% mugwort, 7.3% cockroach, 5.7% *D. pter.*, 5.5% birch, 5.5% cat, 5.3% timothy grass, 3.7% dog according to sIgE measurement	11 components Extract-based No food allergens	Vartiainen et al. 2002 [58]
	20	427 (49)/284	6–16 (12 ± 3)/36 ± 7	random population: children/their mothers	Skin testing + sIgE by UniCAP^®^, Pharmacia Diagnostics (birch, timothy grass, mugwort, cat, dog, horse, *D. pter.*, codfish, egg white, wheat, cow’s milk, peanut)	Children: sIgE—3.8% birch, 5.2% timothy grass, 5.2% mugwort, 4.2% cat, 3.5% dog, 11.7% *D. pter.*, 3.0% wheat; higher rates were obtained by sIgE testing. Mothers: SPT—6.3% birch, 4.6% timothy grass, 8.8% mugwort, 3.9% cat, 5.3% dog, 6.7% *D. pter.*; higher rates were obtained with SPT	12 components Extract-based ISAAC questionnaire No fungi and ragweed allergens	Pekkarinen et al. 2007 [59]
	21	266 (43)	7–15 (11)	random population	UniCAP^®^, Pharmacia Diagnostics: sIgE to chicken egg albumin and allergens of cat and birch	3% egg albumin	3 components only Extract-based	Seiskari et al. 2007 [60]
	22	305 (39)	24–54	random population	UniCAP^®^, Pharmacia Diagnostics: sIgE to allergens of birch, timothy grass, cat, *D. pter.* and *C. herbarum*	14.4% HDM, 3.9% cat, 3.6% timothy grass, 3.3% birch	5 components Extract-based No weed and food allergens	Laatikainen et al. 2011 [61]
Saint Petersburg and Leningrad region	23	642/449/429/126/165	1–86 (24 ± 15)/2–75 (30 ± 15)/2–75 (30 ± 17)/10–69 (36 ± 14)/2–69 (33 ± 16)	AD/AR/atopic BA/HAE/urticaria	ELISA: sIgE to fungi (*P. notatum*, *C. herbarum*, *A. fumigatus*, *M. racemosus*, *C. albicans*, *A. alternata*), food (cow’s milk, codfish, wheat, soy, hazelnut, tomatoes, pork, beef, carrot, strawberry, apple, chicken meat, egg), tree (alder, birch), grass (timothy grass, meadow fescue, rye) and weed (mugwort, dandelion, goosefoot) pollen, HDM (*D. pter.*, *D. far.*), cockroach, house dust and animal (cat and dog dander, chicken feather)	Similar pattern for AR and BA patients (cat, dog and HDM > 50%; tree pollen > 30%; timothy grass > 30%). Urticaria patients exhibited the highest reactivity to *D. pter.* (50%) followed by cat and dog allergens (40%). HAE patients were mostly sensitized to HDM, cat and dog (>40%) followed by grass and weed allergens (>30%). In atopic dermatitis patients, sensitization rate was higher and exceeded 15% for all the allergens tested, most notably higher for fungi (>30%) and food allergens; most frequently recognized were HDM, cat a dog allergens (>60%) and tree pollen (>40%).	35 components Extract-based	Aak et al. 2013 [62]
	24	360 (60)	5–17 (9 ± 3)	AD, BA	Skin testing to house dust, *D. pter.*, *D. far.*, cat, dog, horse, pillow feather allergens	70.3% *D. far.*, 60.8% *D. pter.*, 54.2% cat and 52.8% dog	6 components Extract-based Indoor allergens only	Trusova et al. 2021 [63]
	25	93 (26)	adults (51 ± 16)	severe BA	ELISA: sIgE to birch, timothy grass, mugwort, *A. fumigatus*, *D. pter.*, *D. far.*, house dust, cat and dog	66.3% house dust, 60.0% *D. pter.*, 57.7% *D. far.*, 55.8% cat, 43.2% dog, 41.1% birch, 41.1% mugwort, 38.9% timothy grass pollen	10 components Extract-based No food allergens	Kozlova et al. 2023 [64]
	13	80 (50)	2–8 (5)	allergic	Protia Allergy-Q^®^, ProteomeTech: sIgE by enzyme immunoassay	Cat was the most prominent (42.86% of indoor allergens sensitizations) among indoor allergens, birch—of pollen (53.86% of pollen sensitizations) and milk—of food allergens (44.74% of food sensitizations)	43 components Extract-based	Borisov et al. 2024 [52]
**Southern Federal District**								
Rostov region	26	35 (49)	27–49	seasonal AR	Skin testing to ragweed, mugwort, cyclachena, sunflower and goosefoot allergens	Ragweed 82.1%, cyclachena 65.3%, mugwort 54.6%, sunflower 38.2%	5 components Extract-based Weeds only	Trofimenko et al. 2015 [65]
	13	72 (47)	3–9 (5)	allergic	Protia Allergy-Q^®^, ProteomeTech: sIgE by enzyme immunoassay	Birch was the most notable of pollens (45.45% of pollen sensitizations), milk and egg—of foods (20.69% of food sensitizations each), no dominance in indoor allergens	43 components Extract-based	Borisov et al. 2024 [52]
	27	166 (58)	18–67 (38 ± 16)	atopic BA	ALEX2^®^, MacroArrayDX: sIgE to 178 allergen molecules and 117 allergen extracts	Most notable 43.98% Amb a 1 and 23.49% Art v 1 in weeds, 30.72% Phl p 1 and 28.92% Cyn d 1 in grass, 30.72% Cry j 1 and 22.89% Bet v 1 in tree, 30.12% Fel d 1 in indoor, 16.27% Alt a 1 in fungi	295 components Mixed	Churyukina et al. 2025 [66]
Astrakhan region	28	450	17+	seasonal AR (self-reported)	Skin testing to ash, maple, timothy grass, cock’s foot, ryegrass, hemp, goosefoot, ragweed, mugwort and cyclachena allergens	64.0% goosefoot, 53.8% mugwort, 35.6% cyclachena, 35.1% hemp, 34.67% ragweed, 20.4% maple, 17.1% timothy grass, 15.6% ryegrass, 14.7% cock’s foot, 14.2% ash	10 components Extract-based Pollen only	Shamgunova et al. 2010 [67]
Volgograd region	29	112	18–40	seasonal AR with or without UGITID	Skin testing: alder, birch, hazel, oak, ash, maple, cock’s foot, fescue, timothy, corn, brome, meadow-grass, foxtail, rye, ryegrass, couch grass, bent, mugwort, cyclachena, ragweed, dandelion, goosefoot pollen allergens	Most prominent to alder (36.1%) among tree pollen allergens, cock’s foot (52.6%) among grass pollen allergens and mugwort (80.4%) among weed pollen allergens	23 components Extract-based Pollen only	Iraklionova et al. 2020 [68]
**North Caucasian Federal District**								
Republic of Ingushetia	30	30	18–55 (36 ± 12)	AD, AR, FA in IC or helmintosis patients	ImmunoCAP^®^, Phadia: sIgE to allergens Amb a 1, Art v 1, Der f 1, Der p 1, Bet v 1, Aln g 1, Cor a 1, Phl p 1, Lol p 1, Sec c_pollen, Bos d 4, Gal d 1, Gal d 5, Tri a 14, Mus a, Ory s	30% Amb a 1, 24% Art v 1, 22% Der f 1, 18% Der p 1, 17% Bet v 1, 15% Aln g 1, 14% Cor a 1 in patients sensitized to respiratory allergens (*n* = 23) and 57% Bos d 4, 43% Gal d 1 and Gal d 5, 43% Tri a 14, 29% Mus a and Ory s in FA patients (*n* = 7)	16 components Molecular-based No animal and fungi allergens	Pugoeva et al. 2025 [69]
Republic of North Ossetia-Alania	31	440	18–65	AR	Skin testing to tree (birch, poplar, alder, hazel, oak), weed (mugwort, ragweed, goosefoot), timothy grass pollen as well as fungal (*Cladosporium*, *Alternaria*, *Aspergillus*) allergens	Mainly to weeds (61.4%), grasses (20.9%) and fungal allergens *Alternaria* and *Cladosporium* (22.5%)	12 components Extract-based Pollen and fungi only	Brtsieva et al. 2014 [70]
Republic of Dagestan	32	98 (40)	39.8 ± 4.3	atopic BA	Skin testing: alder, birch, hazel, oak, ash, maple, cock’s foot, fescue, brome, meadow-grass, foxtail, rye, couch grass, mugwort, ragweed, dandelion, goosefoot pollen allergens; sheep, cat, dog, human hair, horse dander, pillow feather, house and storage dust	50.0% house dust, 47.7% sheep hair, 23.9% storage dust, 22.7% cat hair, 15.9% human hair, 12.8% mugwort, 11.4% dog hair, 26.1% pillow feather, 10.1% ragweed, 10.1% goosefoot	25 components Extract-based No fungi and allergens	Gadzhieva et al. 2016 [71]
Chechen Republic	33	845 (41)	4–68 (29)	allergic	Skin testing to tree (mix), grass (mix), weed (mugwort, ragweed) pollen, indoor (cat, dog and horse dander, cockroach, *D. pter.*, *D. far.*) and fungi (*C. herbarum*, *A. fumigatus*, *Alternaria*) allergens	26.6% ragweed, 20.7% mugwort, 52.5% HDM, 51.2% grass mix, 14.9% tree mix	13 components Extract-based No food allergens	Macharadze et al. 2017 [72]
	34	80 (56)	7–8 and 3–14	random population	ImmunoCAP^®^, Phadia: sIgE to *D. pter.*, *D. far.*, *B. tropicalis*, birch, timothy grass, ragweed, mugwort, goosefoot, plantain, cat, dog, *Alternaria*, egg white, cow’s milk, wheat, buckwheat, soy, peanut	38.8% ragweed, 33.8% *D. far.*, 31.3% *D. pter.*, 23.8% timothy grass, 11.3% goosefoot, 11.3% cat, 8.8% wheat, 7.8% buckwheat, 6.3% soy, 6.3% plantain, 6.3% mugwort	13 components Extract-based ISAAC questionnaire	Ibisheva et al. 2022 [73]
**Volga Federal District**								
Orenburg region	18	268	adults	allergic	Skin testing to birch, alder, hazel, timothy grass, cock’s foot, fescue, brome, meadow-grass, foxtail, rye, perennial ryegrass, couch grass, corn, mugwort, goosefoot, dandelion, sunflower, ragweed allergens	Most prominent to alder (17.1%) among tree pollen allergens, cock’s foot (15.7%) among grass pollen allergens and mugwort (57.5%) and ragweed (36.9%) among weed allergens	18 components Extract-based Pollen allergens only	Vahnina et al. 2012 [57]
Nizhny Novgorod region	35	98 (70)	3–17 (9 ± 5)	atopic BA	Skin testing to house dust, storage dust, HDM, cat, dog, horse, pillow feather, tree pollen, grass pollen, weed pollen allergens	72% pollen allergens (58% tree pollen, 43% grass, 39% weeds), 71% dust allergens (56% house dust, 42% HDM, 24% storage dust), 38% animal allergens (30% cat, 16% horse, 15% pillow feather, 6% dog)	10 components Extract-based No food and fungi allergens	Nilova et al. 2019 [74]
Penza region	36	872/1162	7–14/20–60	self-reported AR	Skin testing to house dust, storage dust, cat, dog, *D. pter.*, *D. far.*, grass pollen, weeds, tree pollen, pillow feather, barn mites, sheep, rabbit, cockroach allergens	Children/adults: house dust (12.8/9.7%), storage dust (13.8/7.4%), cat (11.5/9.8%), dog (8.9/5.5%), *D. pter.* (9.5/6.8%), *D. far.* (7.2/7.9%), grass pollen (6.4/15.8%), weeds (6.3/10.1%), tree pollen (7.7/10.3%), pillow feather (3.8/4.2%), barn mites (2.3/4.6%), sheep (3.3/3.1%), rabbit (4.5/3.0%)	14 components Extract-based No food and fungi allergens ISAAC/ECRHS questionnaire	Manzhos et al. 2008 [75]
Republic of Tatarstan	37	24 (50)/20 (40)	4–60 (18 ± 3)/ 3–43 (28 ± 4)	seasonal AR/ perennial AR	ImmunoCAP^®^, Phadia: sIgE to *D. pter.*, *D. far.*, *Der p 1*, *A. alternata*, *C. herbarum*, *R. nigricans*, *P. notatum*, *A. fumigatus*, *A. terreus*, *F. moniliforme*, Bet v 1, Bet v 2, mugwort, SEA, SEB	Seasonal AR group Bet v 1 (95.8%), Bet v 2 (58.3%), mugwort (50.0%), SEB (50.0%), SEA (29.2%) *C. herbarum* (16.7%), *A. fumigatus* (16.7%), *P. notatum* (12.5%); in perennial AR group *D. pter.* (75.0%), *D. far.* (70.0%), SEB (40.0%), SEA (35.0%)	14 components Mixed No grass, animal and food allergens	Tyurin et al. 2018 [76]
Udmurtian Republic	38	154	6–17	seasonal allergy	Skin testing + sIgE by ImmunoCAP^®^, Phadia to tree, grass and weed pollen allergens	79.2% tree pollen (birch, alder, hazel), 73.4% grass pollen (cock’s-foot, fescue, ryegrass) and 20.1% weeds (mugwort, sunflower)	8 components Extract-based Pollen allergens only	Matveeva et al. 2021 [77]
Republic of Bashkortostan	39	60/21	15–55	seasonal allergy/control	Skin testing + sIgE (ELISA) to birch, grass, weed pollen, HDM, dust, fungal, bacterial and cat allergens	Most prominent to oak (15%) among tree pollen allergens, rye (41.7%) among grass pollen allergens and ragweed (31.7%) among weed allergens; 20.0% *A. flavus* and 15.0% *D. far.* Discrepancies with skin testing results	50 components Extract-based No food allergens	Enikeyev et al. 2008 [78]
	40	100 (41)	15–55	seasonal allergy	Skin testing to tree (birch, alder, hazel), grass (timothy grass, cock’s foot, fescue, brome, meadow-grass, meadow foxtail, rye, perennial ryegrass, couch grass, corn), weed (mugwort, goosefoot, dandelion, sunflower, ragweed) pollen allergens	Sensitizations: most prominent to birch (34%) among tree pollen allergens, cock’s foot and ryegrass (42%) among grass pollen allergens and couch grass (38%) among weed pollen allergens	24 components Extract-based Pollen allergens only	Fayurshin et al. 2008 [79]
	41	36 (58)	2–4 (3.1 ± 0.8)	AR	sIgE by ImmunoCAP^®^, Phadia to HDM *D. pter.* and *D. far.*; birch, timothy grass and mugwort pollen; cat and dog dander	Cat dander 61.1%, birch pollen 50.0%, dog dander 44.4%, mugwort 38.9%, HDM 30.6%, *D. pter.* 27.8%, *D. far.* 25.0%, timothy grass 25.0%	7 components Extract-based No fungi and ragweed allergens Pooled food allergen data	Andronova et al. 2022 [80]
Samara region	42	106 (71)/80 (65)	3–17	Urban/rural children with BA	Skin testing to household, epidermal, mold, weed, grass and tree pollen allergens	Household allergens: urban 81.1%/rural 30.0%; tree pollen: 33.0/40.0; epidermal allergens: 26.4%/15%; weed pollen: 22.6%/25.0%; grass pollen: 17.0/25.0; fungal allergens: 11.3%/3.0%	Extract-based No food allergens	Kulagina et al. 2019 [81]
	43	969 (56)	adults	AR	Skin testing to household, pollen, and epidermal allergens	Out of all positive skin test reactions, 50.6% were caused by pollen, 34.9% by household allergens and 14.5% by epidermal allergens	Extract-based Grouped results only No food and fungi allergens	Zhukova et al. 2020 [82]
	44	494 (50)	3–17 (7.7 ± 3.8)/>18 (43.1 ± 14.2)	BA, AR	sIgE by ImmunoCAP^®^, Phadia (Alt a 1, Art v 1, Amb a 1) and RIDA Allergy-screen^®^, R-Biopharm	Pollen sensitization: adults 38%/children 23%; fungal sensitization: adults 18%/children 28% with a 52% total prevalence of sensitization to *A. alternata*	8 components Mixed No food, animal and household allergens	Mazokha et al. 2021 [83]
**Ural Federal District**								
Sverdlovsk region	45	14 (79)	1–12 (6.8)	FA/anaphylaxis	sIgE: ImmunoCAP^®^ ISAC, Phadia	Main sensitizers: Bet v 1 79%, Fel d 1 64%, Can f 1 50%, Milk 43%, egg 43%, Cup a 1 36%, kiwi 21%	112 components Molecular-based Results are partially pooled by source	Lepeshkova et al. 2019 [84]
Chelyabinsk region	46	181 (52)	27–53 (40)	atopic BA	Skin testing with pollen allergens, cat hair, dog hair, house dust, *D. pter.*, storage dust, pillow feather	Pollen 74%, house dust 66%, storage dust 41%, *D. pter.* 21%, feather pillow (12%); epidermal allergens 12%	7 components Extract-based No food and fungi allergens	Zhorina et al. 2020 [85]
	41	71 (59)	2–4 (3.2 ± 0.7)	AR	sIgE by ImmunoCAP^®^, Phadia to HDM *D. pter.* and *D. far.*; birch, timothy grass and mugwort pollen; cat and dog dander	Cat dander 64.8%, dog dander 52.1%, birch pollen 47.8%, mugwort pollen 23.5% and timothy grass pollen 20.3%	7 components Extract-based No fungi and ragweed, pooled food allergen data	Andronova et al. 2022 [80]
	13	58 (21)	6–10 (6)	allergic	Protia Allergy-Q^®^, ProteomeTech: sIgE by enzyme immunoassay	Cat was the most prominent (42.39% of indoor allergen sensitizations) among indoor allergens, birch—of pollen (26.47% of pollen sensitizations) and milk—of food allergens (28.57% of food sensitizations)	43 components Extract-based	Borisov et al. 2024 [52]
**Siberian Federal District**								
Altai Krai	47	128 (70)	3–6 (4.7 ± 1.2)	BA	Skin testing (*D. pter.*, cat and dog dander, birch pollen, grass pollen mix, mugwort pollen, cow’s milk, chicken egg) and sIgE by ImmunoCAP^®^, Phadia	*D. pter.* 42.9%, birch 34.3%, cat dander 25.7%, grass pollen 17.8%, mugwort pollen 13.2%, dog dander 10.9%, chicken egg 10.1%	8 components Extract-based No ragweed and fungi ISAAC questionnaire	Shakhova et al. 2019 [86]
Krasnoyarsk Krai	48	32/38	2–16	FA + AR/BA	Skin testing with chicken egg, cow’s milk, chicken meat, wheat flour, cereals, vegetables and fruits, citrus fruits, fish	Chicken egg 80% AR/73% BA, cow’s milk 62%/68%, cereals 48%/50%, citrus fruits 44%/36%, and fish 49%/11%; also notable was chicken meat 56% for AR and vegetables and fruits 47% for BA	8 components Extract-based Only food allergens	Borisova et al. 2013 [87]
	49	53 (32)/53 (47)	18–66 (33.0 ± 1.3/40.0 ± 1.8)	AD/psoriasis	Skin testing to: *C. albicans*, *A. alternata*, *A. fumigatus*, *C. herbarum*, *P. notatum*, yeast. sIgE by ELISA to the allergen mix: *P. notatum*, *C. herbarum*, *A. fumigatus*, *M. racemosus*, *A. alternata*	*C. albicans* 38.8% AD/48.3% psoriasis, *C. herbarum* 47.4%/62.5%, *P. notatum* 33.3%/33.3%, *A. alternate* 52.2%/52.9%, *A. fumigatus* 35.7%/55.0% and yeast 58.5%/50.9%. Positive sIgE was detected in 15.0% of patients with AD and 5.3% of patients with psoriasis.	7 components Extract-based Only fungal allergens	Barilo et al. 2022 [88]
	50	51 (47)/20 (45)/19	18–57 (40.0 ± 1.8/25.0 ± 2.0)	Psoriasis/AD/control	sIgE by ELISA to cow’s milk, beef, chicken egg, chicken meat, gluten, wheat, oat, rice, buckwheat, potato, carrot, tomato, apple, peanut	Cow’s milk 3.9% psoriasis/25.0% AD, beef 9.8%/10.0%, chicken egg 0%/20%, wheat 2.0%/20.0%, oat 5.9%/35%, buckwheat 11.8%/10.0%, potato 7.8%/0%, carrot 5.9%/10.0%, tomato 2.0%/40.0%, apple 5.9%/30.0%, peanut 2.0%/60.0%. Control group—no sensitizations	14 components Extract-based Only food allergens	Barilo et al. 2024 [89]
	13	182 (41)	3–9.8 (6)	allergic	Protia Allergy-Q^®^, ProteomeTech: sIgE by enzyme immunoassay	Cat was the most prominent (41.89% of indoor allergen sensitizations) among indoor allergens, birch—of pollen (40.68% of pollen sensitizations) and milk—of food allergens (33.33% of food sensitizations)	43 components Extract-based	Borisov et al. 2024 [52]
Far North	13	107 (48)	2–10 (5)	allergic	Protia Allergy-Q^®^, ProteomeTech: sIgE by enzyme immunoassay	Cat was the most prominent (48.39% of indoor allergen sensitizations) among indoor allergens, birch—of pollen (12.0% of pollen sensitizations) and milk—of food allergens (23.83% of food sensitizations)	43 components Extract-based	Borisov et al. 2024 [52]
Tomsk region	51	58 (55)/19 (79)	7–10	EFFA/LFFA	Skin testing and sIgE tested by ImmunoCAP^®^, ImmunoCAP^®^ ISAC to chicken egg, cow’s milk, fish, apple, peach, carrot, celery, tomato, kiwi, melon, peanut, hazelnut	The most prevalent food allergens are egg, fish, apple, carrot in children with both EFFA (10–20%) and LFFA (up to 5%).	12 components Mixed Food allergens only	Evdokimova et al. 2013 [90]
	52	318	7–10	sIgE-positive FA	Skin testing to pollen and food allergens, sIgE by ImmunoCAP^®^, Phadia to peanut (Ara h 1, Ara h 26, Ara h 34, Ara h 8), hazelnut (Cor a 1, Cor a 8, Cor a 11) and birch (Bet v 1)	PR-10 proteins: 21% Bet v 1, 12% Ara h 8, 9% Cor a 1; cupins Ara h 1, Ara h 34, Cor a 11 < 3%; prolamins Ara h 26 and Cor a 8 < 4%; by SPT in patients with FA to hazelnut and peanut: 85.7% birch pollen sensitization, 57.1% to mugwort pollen, 42.9% to weed pollen mix	>13 components Extract-based Food and pollen allergens only	Fedorova et al. 2014 [91]
	53	652/637 (48)	7–10 (8.9 ± 1.1)	FA/control	sIgE tested by ImmunoCAP^®^: 25 foods, HDM, cat; birch, grass, mugwort and parietaire pollen	8.4% HDM, 5.4% cat, 3.3% mugwort, 3.7% birch pollen (aeroallergens), 3.2% hazelnut. sIgE was considered positive with a cutoff > 0.7 kU/L	25 allergens Extract-based No fungi, ragweed	Li et al. 2019 [92]
Novosibirsk region	54	112	24–58	BA	Skin testing to *D. pter.*, *D. far.*, *A. tenius*, *C. herbarum*, *R. nigricans*, *P. notatum*, *A. fumigatus*, *A. terreus*, *F. moniliforme*, *B. cinerea*, cat and dog dander, weed, tree and grass pollen	63% household allergens, 44% animal allergens, 54% pollen, 52% fungi allergens	14 components Extract-based No food allergens	Lazutkina et al. 2011 [93]
	55	25/25/25	18–45	Dishydrotic eczema/AD/control	sIgE by RIDA AllergyScreen^®^, Panel 4, RBiopharm AG to cow’s milk, α-lactoglobulin, β-lactoglobulin, casein, bovine serum albumin, egg white/yolk, soy, carrot, potato, wheat, hazelnut, peanut	AD group: egg white (20%), casein (16%) and milk (12%). Both comparison groups have shown reactivity to milk and its components as well as egg in a range of 4–12%	13 components Extract-based Food allergens only	Bizunova et al. 2017 [94]
Omsk region	56	968	6 m–18	allergic	sIgE by ImmunoCAP^®^, ELISA including food, pollen and household allergens	House dust was the most prominent (90.0% of indoor allergen sensitizations) followed by animal epithelium (47.8%) among indoor allergens (33.7% of all sensitizations). Trees were the most prevalent of pollen allergens (47.8% of pollen sensitizations)	109 components Extract-based	Sokolova et al. 2024 [95]
Irkutsk region	57	139	4.03 ± 0.3	AD, AR	sIgE by ELISA to food, household and pollen allergens	43.2% cow’s milk proteins, 23.7% egg white (ovalbumin)	36 components Extract-based	Dolgikh et al. 2013 [96]
**Far Eastern Federal District**								
Primorsky Krai, Vladivostok	13	105 (48)	3–9 (5)	allergic	Protia Allergy-Q^®^, ProteomeTech: sIgE by enzyme immunoassay	House dust was the most prominent (35.3% of indoor allergen sensitizations) among indoor allergens, birch—of pollen (35.0% of pollen sensitizations) and milk—of food allergens (27.78% of food sensitizations)	43 components Extract-based	Borisov et al. 2024 [52]
Khabarovsk territory	13	82 (58)	2–11 (4)	allergic	Protia Allergy-Q^®^, ProteomeTech: sIgE by enzyme immunoassay	Cat was the most prominent (46.6% of indoor allergen sensitizations) among indoor allergens, birch—of pollen (36.0% of pollen sensitizations) and milk—of food allergens (52.0% of food sensitizations)	43 components Extract-based	Borisov et al. 2024 [52]
Amur region	58	120 (38)	adults (39.9 ± 1.0)	moderate BA	Skin testing: house/storage dust; pillow feather; HDM; cat, dog, sheep, human hair; birch, rye, grass, mugwort, orache, corn pollen; egg, beef, rye flour, rice, oat, buckwheat, fish, citrus	33.3% house dust, 28.3% storage dust, 25.0% pillow feather, 19.2% HDM, 17.5% corn pollen, 10.8% mugwort pollen, 10.0% cat hair, 10.0% sheep hair	22 components Extract-based No ragweed and fungi allergens	Scheglova et al. 2004 [97]
	54	245	24–58	BA	Skin testing to *D. pter.*, *D. far.*, *A. tenius*, *C. herbarum*, *R. nigricans*, *P. notatum*, *A. fumigatus*, *A. terreus*, *F. moniliforme*, *B. cinerea*, cat and dog dander, weed, tree and grass pollen	67% household allergens, 20% animal allergens, 48% pollen, 54% fungi allergens	14 components Extract-based No food allergens	Lazutkina et al. 2011 [93]
Republic of Sakha	54	86	24–58	BA	Skin testing to *D. pter.*, *D. far.*, *A. tenius*, *C. herbarum*, *R. nigricans*, *P. notatum*, *A. fumigatus*, *A. terreus*, *F. moniliforme*, *B. cinerea*, cat and dog dander, weed, tree and grass pollen	55% household allergens, 61% animal allergens, 47% pollen, 64% fungi allergens	14 components Extract-based No food allergens	Lazutkina et al. 2011 [93]
	59	500	1–5	allergic	RIDA AllergyScreen Panel 23	25% house dust, 20% animal, 35% food, 10% pollen, 6% fungi allergens (of which, *A. alternata* 54%, *A. fumigatus* 25%, *C. herbarum* 16%, *P. notatum* 5%)	20 components Extract-based	Ivanova et al. 2021 [98]
Zabaikalsky Krai	60	130	12–18	asthmatic	Skin testing with house dust; cat, dog, and guinea pig hair; birch, alder, mugwort, fescue, timothy grass, cock’s-foot, hazel pollen	Monovalent sensitization by house dust in 9.1% and by pollen allergens in 21.2%. In pollen sensitizers most prominent were grasses (timothy, fescue, cock’s-foot), weeds (mugwort) and tree pollen (birch, alder)	11 components Extract-based No ragweed, fungi, food ISAAC questionnaire	Batozhargalova et al. 2011 [99]

* N = number of participants; (m) = male, %. ** Age is expressed in years or months where indicated with “m”; (aver.) = average age ± standard deviation (SD) where available. AD = atopic dermatitis, AR = allergic rhinitis, BA = bronchial asthma, *D. pter.* = *D. pteronyssinus*, *D. far.* = *D. farinae*, ELISA = enzyme-linked immunosorbent assay, HAE = hereditary angioedema, HDM = house dust mites, IC = immunocompromised, ISAAC = International Study of Asthma and Allergies in Childhood, SEA = Staphylococcal enterotoxin A, SEB = Staphylococcal enterotoxin B, sIgE = allergen-specific immunoglobulin E, SPT = skin prick test, UGITID = upper gastrointestinal tract inflammatory diseases.

**Table 2:** Diagnostic modalities for IgE sensitization assessment in the assessed studies and the associated measurement bias.

Test Modality Category	Studies	Measurement Bias
1. Skin testing (SPT/scarification/intradermal) using allergen extracts	24 studies (40%) [41,47,51,54,55,56,57,63,65,67,68,70,71,72,74,75,79,81,82,85,87,93,97,99]	Tends to overestimate prevalence because extracts contain cross-reactive proteins; however SPT detects mast-cell-bound IgE and can be positive even when serum IgE is low or absent.
2. Extract-based serology (ImmunoCAP extracts)	9 studies (15%) [42,44,46,59,60,61,73,80,92]	Moderately overestimates prevalence. Influenced by cross-reactive extract material, but less sensitive than SPT; misses tissue-bound IgE.
3. sIgE for single allergens (ImmunoCAP^®^, ImmunoCAP^®^ ISAC, MeDALL)	3 studies (5%) [45,50,84]	Does not overestimate prevalence relative to SPT/extracts. Removes cross-reactivity background; identifies true molecular sensitizers; more specific but less sensitive for broad extract reactivity.
4. Mixed extract-based SPT/sIgE with molecular CRD (e.g., ALEX^2®^)	11 studies (18%) [48,49,53,58,66,69,76,77,86,90,91]	Balanced profile if both panels are comprehensive. Extracts inflate prevalence; CRD deflates it; useful for distinguishing genuine vs. cross-reactive sensitization.
5. Experimental assays (custom ELISA, hydrogel biochips, RIDA AllergyScreen^®^, Polycheck^®^, Protia Allergy-Q^®^)	13 studies (22%) [40,43,52,62,64,78,83,88,89,94,95,96,98]	Unpredictable bias, often upward due to poor standardization and non-specific binding.

CRD = component-resolved diagnostics, ELISA = enzyme-linked immunosorbent assay, sIgE = allergen-specific immunoglobulin E, and SPT = skin prick test.

## Data Availability

No new data were created or analyzed in this study. Data sharing is not applicable to this article.

## Supplementary Materials

The following supporting information can be downloaded at: https://www.mdpi.com/article/10.3390/ijms27125334/s1.

## Abbreviations

The following abbreviations are used in this manuscript:

AIT	Allergen-specific immunotherapy
AD	Atopic dermatitis
APC	Antigen-presenting cell
AR	Allergic rhinitis
BA	Bronchial asthma
CAP	Carrier polymer system (ImmunoCAP)
CCDs	Cross-reactive carbohydrate determinants
ECRHS	European community respiratory health survey
EFFA	Early-onset food allergy
ELISA	Enzyme-linked immunosorbent assay
FA	Food allergy
FcεR	Fc epsilon receptor
FcεRI	High-affinity Fc epsilon receptor I
FD	Federal district
GRADE	Grading of Recommendations, Assessment, Development and Evaluations
HAE	Hereditary angioedema
HDM	House dust mite
HLA	Human leukocyte antigen
IC	Immunocompromised
Ig	Immunoglobulin
IL	Interleukin
ISAAC	International study of asthma and allergies in childhood
JBI	Joanna Briggs Institute
LFFA	Late-onset food allergy
MeDALL	Mechanisms of the development of allergy
MeSH	Medical subject headings
MHC	Major histocompatibility complex
nsLTP	Non-specific lipid transfer protein
PR-10	Pathogenesis-related protein family 10
PRISMA	Preferred reporting items for systematic reviews and meta-analyses
PROSPERO	International prospective register of systematic reviews
QGIS	Quantum geographic information system
SEA	Staphylococcal enterotoxin A
SEB	Staphylococcal enterotoxin B
sIgE	Specific immunoglobulin E
SPT	Skin prick test
SWiM	Synthesis without meta-analysis
TCR	T-cell receptor
Th2	T helper type 2 cell
UGITID	Upper gastrointestinal tract inflammatory diseases
