# Supplementary material for: Towards Sensitization Profiling for Allergy Prevention in Russia: A Systematic Review
Source: Int J Mol Sci. 2026 Jun 12;27(12):5334. doi: 10.3390/ijms27125334 (PMC13300535; doi:10.3390/ijms27125334)
Supplement: Supplementary file 1 [file ijms-27-05334-s001.zip › ijms-4207350-supplementary.pdf]

Appendix: Supplementary data

Figure S1: Joanna Briggs Institute (JBI) risk of bias assessment for all included studies. (A) Traffic-light plot for studies 1–30 and (B) for studies 31–60. (C) Summary plot aggregating the proportion of studies rated as low, unclear, or high risk of bias for each JBI domain across the full set of 60 studies. The image was generated using the robvis visualization tool, showing domain-level judgments across all JBI appraisal items.

Figure S2: Certainty of evidence assessed using the GRADE framework for all included studies. (A) Studies 1-30 and (B) studies 31-60. Each row represents an individual study, and each column corresponds to a GRADE domain, including risk of bias, inconsistency, indirectness, imprecision, publication bias, and overall certainty of evidence. Color-coded symbols indicate the judgment for each domain: green - not serious/high; yellow - serious/moderate; orange - low; red - very low/very serious. The plot was created using the Critiplot tool.

Table S1. Subgroup and sensitivity summaries of allergen sensitization prevalences in Moscow and Moscow region studies

Allergen	Number of studies	Mean (%)	Median (%)	SD (%)	Min (%)	Max (%)	Median (standard)	Δ median (abs)	Δ median (%)	Pediatric / adult studies	Allergen panels (Full / Restricted)	Modalities reporting (experimental; other; molecular)
Birch	5	58.36	63.10	21.81	30.00	80.56	69.62	+6.52	+10.3%	both pediatric & adult	Full:1 / Restricted:4	1; 2; 2
Grass	3	31.39	39.77	18.67	10.00	44.40	42.09	+2.31	+5.8%	mostly pediatric/mixed	Full:1 / Restricted:2	1; 2; 0
Weed*	1	55.68	55.68	-	55.68	55.68	55.68	0.00	0.0%	pediatric	Full:0 / Restricted:1	0; 1; 0
Cat	3	35.70	25.00	23.92	19.00	63.10	41.05	+16.05	+64.2%	both pediatric & adult	Full:0 / Restricted:3	1; 1; 1
House dust mite	2	48.47	48.47	23.38	31.94	65.00	31.94	−16.53	−34.1%	mixed	Full:1 / Restricted:1	1; 1; 0
Milk	3	18.27	15.00	8.39	12.00	27.80	19.90	+4.90	+32.7%	predominantly pediatric	Full:0 / Restricted:3	1; 1; 1
Egg	3	23.67	18.00	12.50	15.00	38.00	28.00	+10.00	+55.6%	predominantly pediatric	Full:0 / Restricted:3	1; 1; 1
Peanut	2	36.00	36.00	15.56	25.00	47.00	47.00	+11.00	+30.6%	predominantly pediatric	Full:0 / Restricted:2	1; 0; 1

*Single study evidence; allergen arrays include weed allergens but reported no relevant sensitization in studies populations.

Number of studies is reported per allergen. Medians, means, standard deviations and ranges are shown; "median (standard)" is the median after excluding experimental platforms (custom ELISA, hydrogel biochips, RIDA AllergyScreen®, Polycheck®, Protia Allergy-Q®).

A

Study	D1	D2	D3	D4	D5	D6	D7	D8	D9	Overall
1. Mokronosova et al. 2003	X	X	X	-	X	-	+	-	?	X
2. Akhapkina et al. 2014	X	X	-	-	X	+	+	-	?	X
3. Snovskaya et al. 2016	X	X	+	-	X	+	+	+	?	-
4. Voloshin et al. 2018	X	X	+	+	-	-	+	+	?	-
5. Yasakov et al. 2019	X	X	-	-	X	+	+	+	?	X
6. Elisyutina et al. 2020	X	-	+	+	X	+	+	+	?	-
7. Levina et al. 2021	-	X	+	+	X	+	+	-	?	-
8. Smolkin et al. 2022	X	X	-	-	X	+	+	-	?	X
9. Mokronosova et al. 2023	X	X	-	-	X	+	+	+	?	X
10. Zheltikova et al. 2024	-	X	+	+	-	+	+	+	?	-
11. Fomina et al. 2024	X	X	-	-	X	+	+	-	?	X
12. Akhapkina et al. 2024	X	X	-	-	X	+	+	-	?	X
13. Borisov et al. 2024	X	-	-	-	X	+	+	-	?	X
14. Efendieva et al. 2024	X	X	+	+	X	+	+	-	?	-
15. Vorontsova et al. 2018	X	X	-	-	X	-	+	-	?	X
16. Bergets et al. 2007	X	X	-	-	X	+	+	-	?	X
17. Bekezin et al. 2020	X	X	-	-	X	-	+	-	?	X
18. Vahnina et al. 2012	-	X	+	-	-	+	+	-	?	X
19. Vartiainen et al. 2002	+	+	+	+	+	+	+	+	-	+
20. Pekkarinen et al. 2007	+	+	+	+	+	+	+	+	-	+
21. Seiskari et al. 2007	+	+	+	+	+	+	+	+	-	+
22. Laatikainen et al. 2011	+	+	+	+	+	+	+	+	-	+
23. Aak et al. 2013	X	X	+	-	X	X	+	-	?	X
24. Trusova et al. 2021	X	X	-	-	X	+	+	-	?	X
25. Kozlova et al. 2023	X	X	-	-	X	X	+	-	?	X
26. Trofimenko et al. 2015	X	X	-	-	X	-	+	-	?	X
27. Churyukina et al. 2025	X	X	-	-	X	+	+	-	?	X
28. Shamgunova et al. 2010	-	-	+	-	X	+	+	-	?	X
29. Iraklionova et al. 2020	X	X	-	-	X	+	+	-	?	X
30. Pugoeva et al. 2025	X	X	-	-	X	+	+	-	?	X

D1: Sample frame
D2: Sampling method
D3: Sample size adequate
D4: Subjects & setting described
D5: Coverage of population
D6: Valid measurement
D7: Same measurement for all
D8: Statistical analysis
D9: Response rate adequate

Judgement: X High; - Unclear; + Low; ? No information

Figure S1

B

Study	D1	D2	D3	D4	D5	D6	D7	D8	D9	Overall
31. Brtsieva et al. 2014	-	X	+	-	X	+	+	-	?	X
32. Gadzhieva et al. 2016	X	X	+	-	X	+	+	-	?	X
33. Macharadze et al. 2017	X	X	-	-	X	+	+	-	?	X
34. Ibisheva et al. 2022	X	-	-	-	X	+	+	-	?	X
35. Nilova et al. 2019	X	X	-	-	X	+	+	-	?	X
36. Manzhos et al. 2008	-	X	+	-	-	+	+	-	?	X
37. Tyurin et al. 2018	X	X	-	-	X	+	+	-	?	X
38. Matveeva et al. 2021	X	X	+	-	X	+	+	-	?	X
39. Enikeyev et al. 2008	X	X	-	-	X	X	+	-	?	X
40. Fayurshin et al. 2008	X	X	-	-	X	-	+	-	?	X
41. Andronova et al. 2022	X	X	-	-	X	X	+	-	?	X
42. Kulagina et al. 2019	X	X	+	-	X	+	+	-	?	X
43. Zhukova et al. 2020	X	X	+	-	-	X	+	-	?	X
44. Mazokha et al. 2021	X	X	+	-	X	+	+	-	?	X
45. Lepeshkova et al. 2019	X	X	-	-	X	+	+	-	?	X
46. Zhorina et al. 2020	X	X	+	-	X	+	+	-	?	X
47. Shakhova et al. 2019	-	-	+	-	X	X	+	-	?	X
48. Borisova et al. 2013	X	X	-	-	X	-	+	-	?	X
49. Barilo et al. 2022	X	X	-	-	X	X	+	-	?	X
50. Barilo et al. 2024	X	X	-	-	X	X	+	-	?	X
51. Evdokimova et al. 2013	X	X	+	-	X	+	+	-	?	X
52. Fedorova et al. 2014	X	X	+	-	X	+	+	-	?	X
53. Li et al. 2019	+	-	+	-	+	+	+	X	X	-
54. Lazutkina et al. 2011	X	X	-	-	X	-	+	-	?	X
55. Bizunova et al. 2017	X	X	-	-	X	+	+	-	?	X
56. Sokolova et al. 2024	X	X	+	-	-	-	+	-	?	X
57. Dolgikh et al. 2013	X	X	-	-	X	X	+	-	?	X
58. Scheglova et al. 2004	X	X	-	-	X	+	+	-	?	X
59. Ivanova et al. 2021	X	X	+	-	X	+	+	-	?	X
60. Batozhargalova et al. 2011	X	X	+	-	X	+	+	-	?	X

D1: Sample frame
D2: Sampling method
D3: Sample size adequate
D4: Subjects & setting described
D5: Coverage of population
D6: Valid measurement
D7: Same measurement for all
D8: Statistical analysis
D9: Response rate adequate

Judgement: X High; - Unclear; + Low; ? No information

Figure S1

C

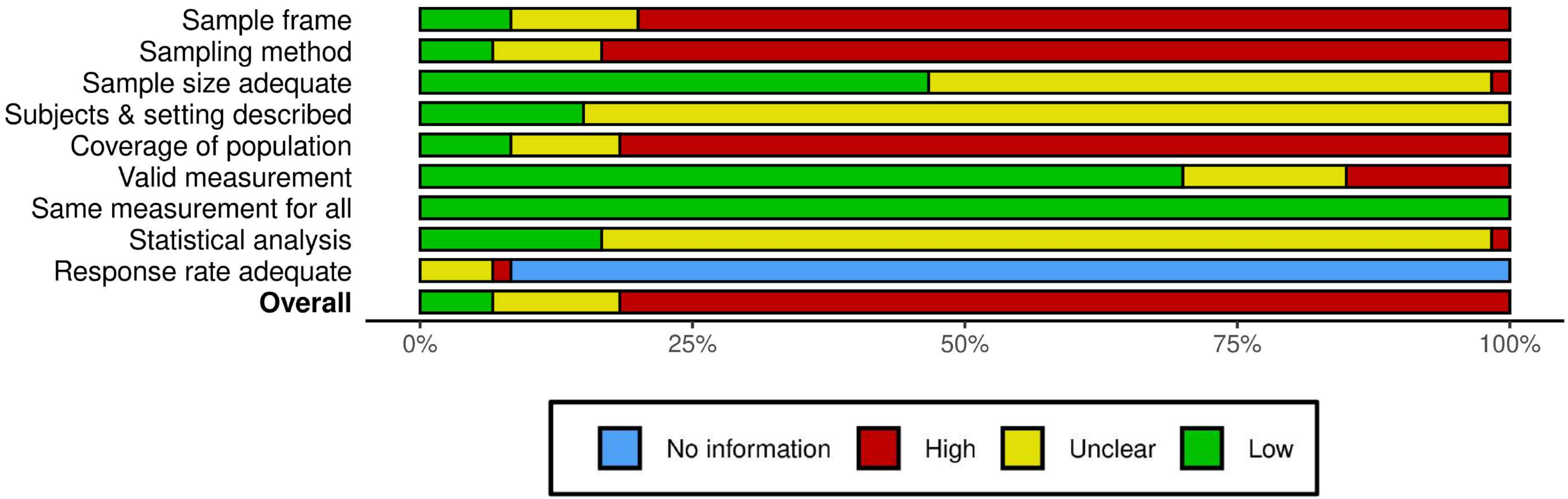
Sample frame
Sampling method
Sample size adequate
Subjects & setting described
Coverage of population
Valid measurement
Same measurement for all
Statistical analysis
Response rate adequate
Overall
0%
25%
50%
75%
100%
No information
High
Unclear
Low

Figure S1

A

GRADE Evidence Profile

	Risk of Bias	Inconsistency	Indirectness	Imprecision	Publication Bias	Overall Certainty
1. Mokronosova et al. 2003	-	+	-	X	+	X
2. Akhapkina et al. 2014	-	+	X	-	+	X
3. Snovskaya et al. 2016	-	+	X	+	+	X
4. Voloshin et al. 2018	-	+	-	+	+	X
5. Yasakov et al. 2019	-	+	X	-	+	X
6. Elisyutina et al. 2020	-	+	+	+	+	-
7. Levina et al. 2021	-	+	-	+	+	X
8. Smolkin et al. 2022	-	+	-	-	+	X
9. Mokronosova et al. 2023	-	+	-	-	X	X
10. Zheltikova et al. 2024	-	+	-	+	X	X
11. Fomina et al. 2024	-	+	-	-	-	X
12. Akhapkina et al. 2024	-	+	-	-	+	X
13. Borisov et al. 2024	-	+	-	-	-	X
14. Efendieva et al. 2024	-	+	-	+	X	X
15. Vorontsova et al. 2018	-	+	-	-	+	X
16. Bergets et al. 2007	-	+	X	-	+	X
17. Bekezin et al. 2020	-	+	X	-	+	X
18. Vahnina et al. 2012	-	+	X	+	+	X
19. Vartiainen et al. 2002	+	+	+	+	+	~
20. Pekkarinen et al. 2007	+	+	+	+	+	~
21. Seiskari et al. 2007	+	+	+	+	+	~
22. Laatikainen et al. 2011	+	+	+	+	+	~
23. Aak et al. 2013	-	+	-	+	+	X
24. Trusova et al. 2021	-	+	X	-	+	X
25. Kozlova et al. 2023	-	+	-	-	+	X
26. Trofimenko et al. 2015	-	+	X	X	+	X
27. Churyukina et al. 2025	-	+	-	-	+	X
28. Shamgunova et al. 2010	-	+	X	+	+	X
29. Iraklionova et al. 2020	-	+	X	-	+	X
30. Pugoeva et al. 2025	-	+	-	X	+	X

Domain Judgments
- Not serious (+)
- Serious (-)
- Very serious (X)
- Not reported (?)

Overall Certainty
- High (+)
- Moderate (~)
- Low (-)
- Very low (x)

1. Risk of Bias: Study design flaws
2. Inconsistency: Results vary across studies
3. Indirectness: Evidence not directly applicable
4. Imprecision: Wide or uncertain estimates
5. Publication Bias: Missing or selective studies
6. Overall Certainty: Confidence in true effect

Figure S2

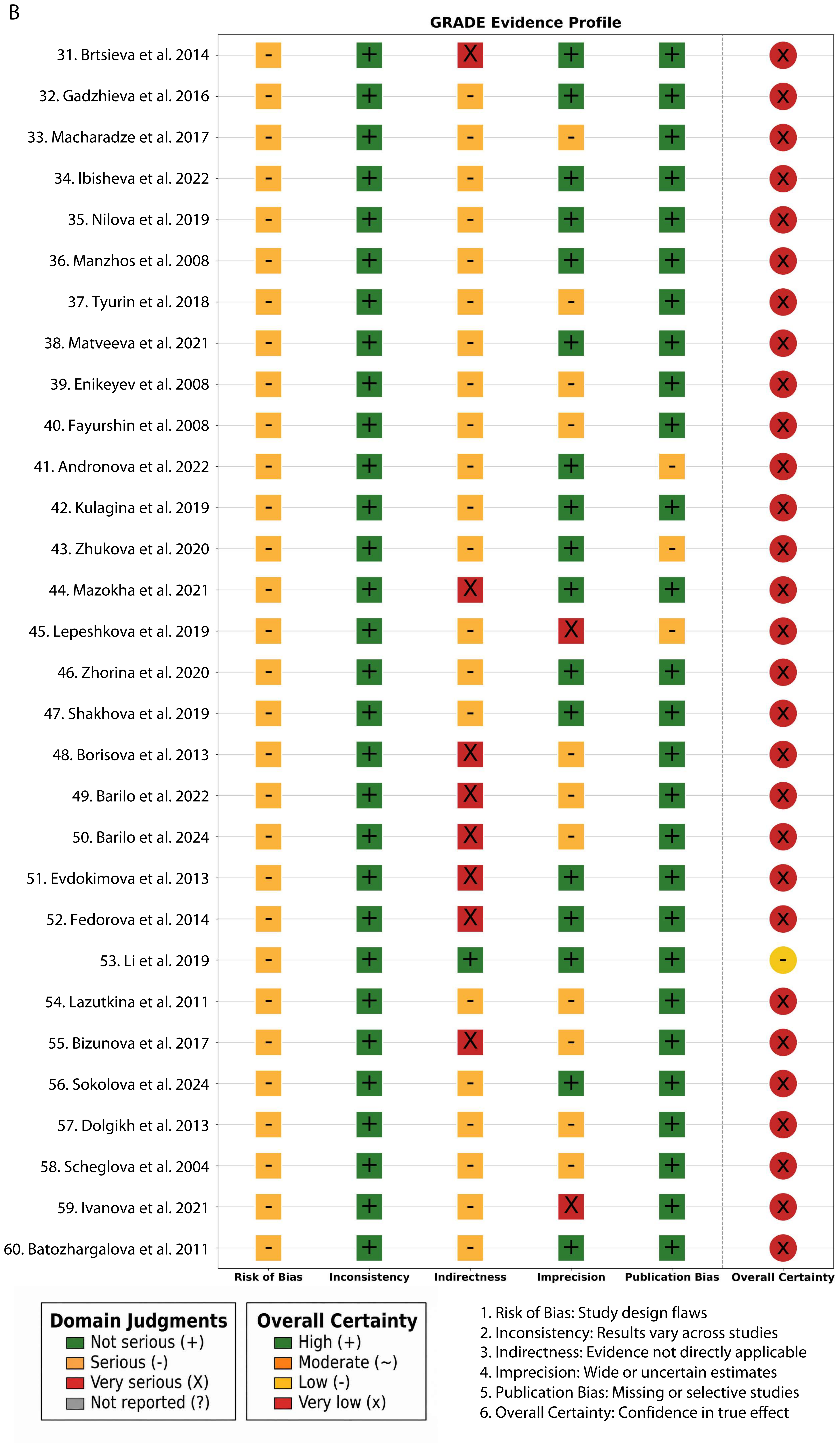
B
GRADE Evidence Profile
31. Brtsieva et al. 2014 - + X + + X
32. Gadzhieva et al. 2016 - + - + + X
33. Macharadze et al. 2017 - + - - + X
34. Ibisheva et al. 2022 - + - + + X
35. Nilova et al. 2019 - + - + + X
36. Manzhos et al. 2008 - + - + + X
37. Tyurin et al. 2018 - + - - + X
38. Matveeva et al. 2021 - + - + + X
39. Enikeyev et al. 2008 - + - - + X
40. Fayurshin et al. 2008 - + - - + X
41. Andronova et al. 2022 - + - + - X
42. Kulagina et al. 2019 - + - + + X
43. Zhukova et al. 2020 - + - + - X
44. Mazokha et al. 2021 - + X + + X
45. Lepeshkova et al. 2019 - + - X - X
46. Zhorina et al. 2020 - + - + + X
47. Shakhova et al. 2019 - + - + + X
48. Borisova et al. 2013 - + X - + X
49. Barilo et al. 2022 - + X - + X
50. Barilo et al. 2024 - + X - + X
51. Evdokimova et al. 2013 - + X + + X
52. Fedorova et al. 2014 - + X + + X
53. Li et al. 2019 - + + + + -
54. Lazutkina et al. 2011 - + - - + X
55. Bizunova et al. 2017 - + X - + X
56. Sokolova et al. 2024 - + - + + X
57. Dolgikh et al. 2013 - + - - + X
58. Scheglova et al. 2004 - + - - + X
59. Ivanova et al. 2021 - + - X + X
60. Batozhargalova et al. 2011 - + - + + X
Risk of Bias
Inconsistency
Indirectness
Imprecision
Publication Bias
Overall Certainty
Domain Judgments
Not serious (+)
Serious (-)
Very serious (X)
Not reported (?)
Overall Certainty
High (+)
Moderate (~)
Low (-)
Very low (x)
1. Risk of Bias: Study design flaws
2. Inconsistency: Results vary across studies
3. Indirectness: Evidence not directly applicable
4. Imprecision: Wide or uncertain estimates
5. Publication Bias: Missing or selective studies
6. Overall Certainty: Confidence in true effect

Figure S2
